# The correlation between serum vitamin D with Apo B and framingham risk score among a group of Iraqi subjects: a Cross-sectional and prospective pilot study

**DOI:** 10.1186/s12872-025-04855-w

**Published:** 2025-07-03

**Authors:** Israa Nather Ahmed, Fatimatuzzahra’ Abd Aziz, Raid Dhia Hashim

**Affiliations:** 1 Discipline of Clinical Pharmacy, School of Pharmaceutical Sciences, Universiti Sains Malaysia, Penang, Malaysia; 2 Pharmacy Department, Al Rasheed University College, Baghdad, Iraq; 3 Discipline of Clinical Pharmacy, School of Pharmaceutical Sciences, Universiti Sains Malaysia, Pulau Pinang, 11800 Malaysia; 4 College of Pharmacy, Al-Farahidi University, Baghdad, Iraq

**Keywords:** Apolipoprotein B, Atherosclerosis, Cardiovascular risk, Framingham risk score, Lipid metabolism, Vitamin D

## Abstract

**Background:**

Vitamin D may play a role in cardiovascular health, particularly in lipid metabolism and atherosclerosis. This study examines the correlation between serum vitamin D levels with Apolipoprotein B (Apo B), and the Framingham Risk Score (FRS) and evaluates the impact of correcting severe vitamin D deficiency on Apo B levels and FRS among a group of Iraqi population.

**Methods:**

This two-phase study was conducted in Baghdad between November 2022 and October 2023 and included a cross-sectional phase examining the association between vitamin D, with Apo B, and the FRS, followed by a prospective phase assessing the impacts of vitamin D correction. A total of 201 participants were recruited, including 60 individuals with severe vitamin D deficiency (≤ 10 ng/ml) who received supplementation and 40 with sufficient vitamin D levels (≥ 30 ng/ml) serving as controls. Levels of total cholesterol (TC), high-density lipoprotein cholesterol (HDL-C), Apo B, and FRS were evaluated at baseline and after six months. The Thai Clinical Trials Registry (TCTR) has retrospectively registered and approved the study under the identification number TCTR20250301003 on the 1st of March 2025.

**Results:**

Vitamin D levels correlated significantly with age (*p* < 0.001), Apo B (*p* = 0.007), and FRS (*p* = 0.003) in the cross-sectional phase. After supplementation TC (*p* = 0.004) and FRS (*p* = 0.007) significantly decreased in the treatment group, with no significant changes in Apo B. Males only showed significant decrease in FRS and TC.

**Conclusion:**

Vitamin D correction significantly decreased TC and FRS reinforcing its role in lipid metabolism and cardiovascular health. However, Apo B levels remained unchanged, suggesting that vitamin D may not directly influence Apo B metabolism in the short term. These findings emphasize the importance of correcting severe vitamin D deficiency before calculating FRS due to its impact on lipid parameters.

**Supplementary Information:**

The online version contains supplementary material available at 10.1186/s12872-025-04855-w.

## Introduction

Most non-communicable diseases deaths are caused by cardiovascular disorders. Every year, 17.7 million people worldwide are affected, mainly in low- and middle-income nations. This necessitates effective risk assessment tools for early intervention [1]. The Framingham Risk Score (FRS) is a well-established predictive model that estimates the 10-year risk of developing CAD and is widely used for primary prevention by identifying high-risk individuals [2]. Over the years, researchers have explored the association between FRS and various biomarkers, including leptin, homocysteine, C-reactive protein, and interleukin-6, in an effort to improve risk stratification. However, an emerging area of interest in cardiovascular research is the potential role of vitamin D in apolipoprotein B (Apo B) and lipid metabolism, and CAD pathogenesis [3, 4].

Traditionally, vitamin D has been primarily recognized for its role in bone health and calcium homeostasis. However, recent evidence suggests a broader physiological impact, particularly in vascular health, lipid metabolism, and inflammation, which are all crucial contributors to CAD development [4, 5]. Vitamin D has been hypothesized to influence insulin sensitivity and secretion, indirectly modulating lipid metabolism. Additionally, it may regulate hepatic triglyceride secretion through its role in calcium homeostasis, further linking vitamin D to cardiovascular risk. Despite these potential mechanisms, the precise biological pathways through which vitamin D influences lipid metabolism and CAD progression remain unclear and actively debated [6, 7].

Recent studies indicate that vitamin D deficiency is associated with an unfavourable lipid profile, including increased Apo B levels, which may contribute to a higher risk of cardiovascular disease [8]. However, the effects of vitamin D supplementation on apolipoproteins remain controversial, with some studies reporting no significant changes in Apo B levels following vitamin D correction [8] ​. Given these inconsistencies, further research is necessary to clarify the potential impact of vitamin D on lipid metabolism and cardiovascular risk modulation.

Observational studies have reported an inverse association between serum vitamin D levels and CAD, suggesting that vitamin D deficiency is linked to increased cardiovascular risk [9]. However, randomized controlled trials (RCTs) have failed to demonstrate a significant decrease in CAD incidence or mortality following vitamin D supplementation [10–12]. This discrepancy raises questions about the true causal association between vitamin D and cardiovascular health, likely influenced by confounding variables such as nutritional status, ethnicity, outdoor physical activity, and the presence of chronic diseases. Moreover, vitamin D supplementation appears to have minimal impact on CAD risk among individuals with adequate baseline vitamin D levels and normal lipid profiles. In contrast, those with severe vitamin D deficiency exhibit an increased CAD risk, possibly due to elevated parathyroid hormone (PTH) levels, which is a highly sensitive indicator of vitamin D deficiency and has established cardiovascular implications [13, 14]. Given these complexities, there is a critical need for targeted investigations that focus specifically on understudied Iraqi individuals with severe vitamin D deficiency to assess the potential cardiovascular benefits of correcting their vitamin D levels.

The current study aims to bridge this research gap by investigating the correlation between serum vitamin D levels with Apo B and FRS and to determine whether correcting severe vitamin D deficiency can influence serum Apo B levels and FRS outcomes. By focusing on an understudied Iraqi population, this study provides novel insights into the association between vitamin D, lipid metabolism, and long-term CAD risk, potentially informing more targeted preventive strategies in clinical practice.

## Methodology

This study was conducted in accordance with the STROBE (Strengthening the Reporting of Observational Studies in Epidemiology) guidelines, ensuring methodological rigor, transparency, and reproducibility in reporting [15]. This study consisted of two phases: a cross-sectional phase to assess the baseline association between serum vitamin D levels with Apo B and the FRS and a prospective phase to evaluate the impact of vitamin D correction in individuals with severe deficiency. This two-phase design enabled us to explore correlations in phase 1 and then confirm causality in phase 2 while reducing confounding, particularly due to age. The study was conducted at Dr. Abas Abd Almuaed’s Clinical Laboratory in Baghdad from November 2022 to October 2023. All subjects attending the aforementioned clinical laboratory for routine vitamin D test were invited to participate in the study after providing their consent.

### Recruitment and selection criteria

**Fig. 1:**
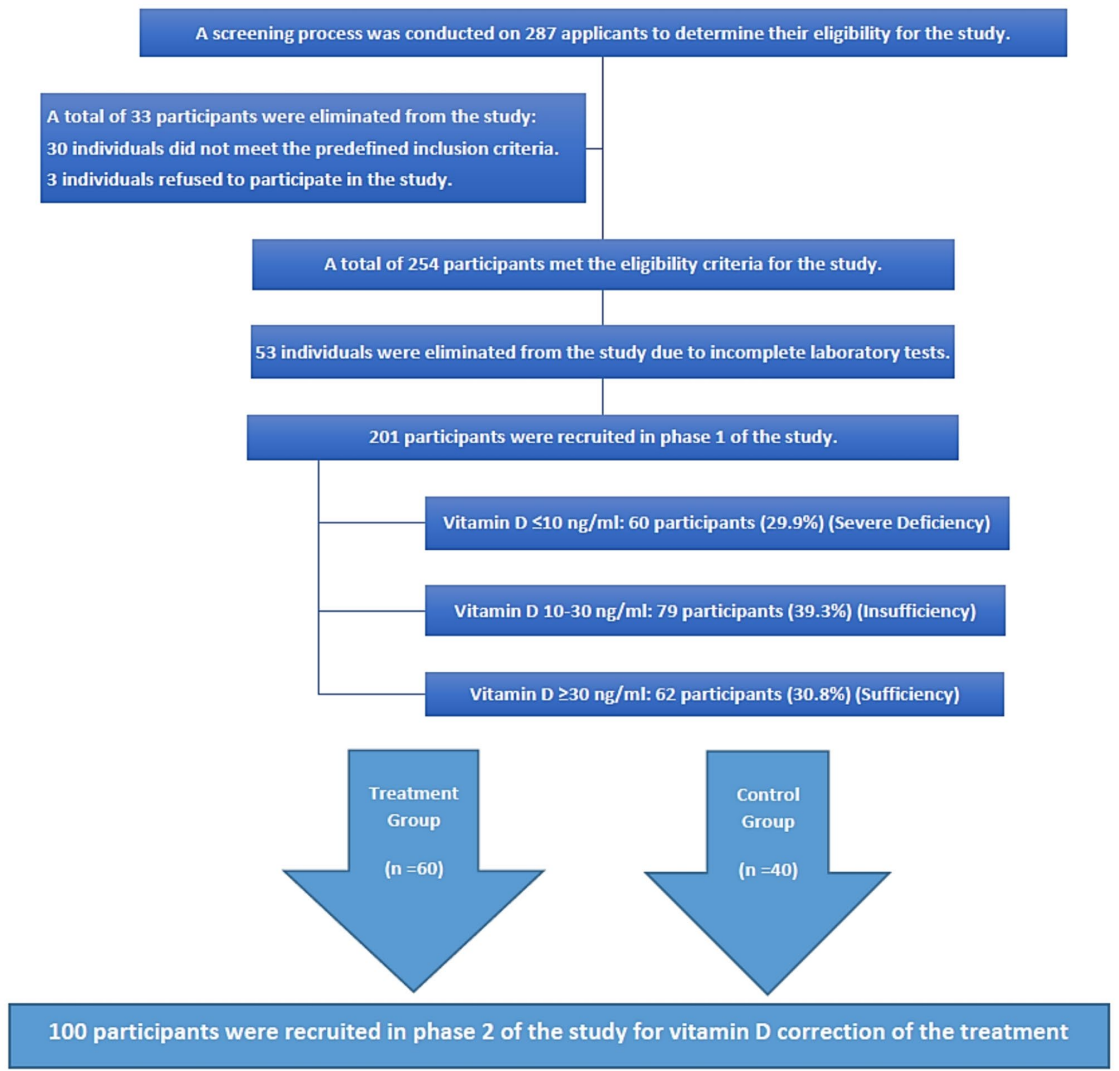
Recruitment Process of the Participants

Eligible participants were adults aged ≥ 30 years, in a fasting state for 8–12 h, and undergoing vitamin D testing at the clinical laboratory. All participants had normal fasting blood glucose, liver function, and renal function tests at screening and provided written informed consent before participation. Exclusion criteria included individuals with a history of cardiovascular disease (except hypertension), chronic or acute illnesses, medication use (except for hypertension), pregnancy, or breastfeeding. Hypertension was identified based on current antihypertensive treatment recorded in medical files. All included participants had blood pressure within the acceptable range at the time of assessment, and no classification was made based on single-visit BP measurements. The minimum sample size for phase 1 was determined using the modified two-mean formula, yielding a target of 120 participants. However, 287 eligible Iraqi participants were selected for the screening. Thirty-three were excluded (15 with diabetes, 2 pregnant, 13 with cardiovascular disease, and 3 who declined participation). After removing 53 participants due to incomplete data, the final sample for phase 1 included 201 participants. For phase 2, the pilot study sample size estimation formula was applied. Sixty participants with severe vitamin D deficiency (≤ 10 ng/ml) were allocated to the treatment group, while 40 participants with sufficient vitamin D levels (≥ 30 ng/ml) formed the control group. For Phase 2, subjects with insufficient but not severely deficient vitamin D levels were excluded. The control group of 40 participants was randomly selected from the 62 subjects in Phase 1 who had sufficient vitamin D levels (≥ 30 ng/ml), using a computer-generated random number function. Figure 1 illustrates the recruitment process of the study participants.

### Ethical considerations

Ethical approval was obtained from the Research and Ethics Committee of Universiti Sains Malaysia (**USM/JEPeM/22060348**) and the Al-Rasheed Research Ethics Committee, Iraq (**RUCPD30122202 and RUCPD30122204**) before study initiation. Informed consent documents were translated into Arabic and provided to all participants before enrollment. Those continuing into phase 2 signed an additional informed consent form.

### Data collection and measurements

Demographic data, including age, sex, smoking status, and antihypertensive therapy use, were retrieved from medical records. Anthropometric and clinical measurements were taken using standardized protocols. Blood pressure (BP) was measured in a seated position using a membrane sphygmomanometer, with two consecutive readings averaged for analysis. Body weight and height were recorded using a digital scale and stadiometer, and body mass index (BMI) was calculated accordingly. Biochemical analyses were conducted within the same laboratory to ensure uniformity. Blood samples (5 mL) were drawn from the antecubital vein of each participant and analyzed for serum vitamin D, TC, HDL-C and Apo B. Serum vitamin D levels were measured using the Chemiluminescent method (Cobas E411 Analyzer, Roche Company), while TC and HDL-C concentrations were determined using enzymatic colorimetric and homogeneous colorimetric assays (Autoanalyzer C311, Roche Company). Lipid profile analysis was conducted on the same day, and the serum sample for Apo B analysis was stored in the freezer at – 20C^0^ to be analyzed collectively within a month. Serum Apo B was assessed by the Human Apo B ELISA Kit (BT LAB Human Apolipoprotein B, apo-B ELISA Kit) (ab 190806) which is a single-wash 90-minute sandwich ELISA intended for the quantitative measurement of serum Apo B. Additionally, the FRS for each participant was calculated using an Excel-based computerized tool (https://qxmd.com/calculate/calculator_253/framingham-risk-score-atp-iii), incorporating relevant cardiovascular risk factors such as age, sex, TC, HDL-C, systolic BP, antihypertensive medication use, and smoking status [16]. The excess blood specimen was treated by an autoclave and daily collected in special containers by a representative of the Ministry of Health of Iraq where the tainted samples were disposed of according to the Iraqi laboratory guidelines. All instruments were validated by daily quality control and calibration as needed; tools were validated by calibration as required.

Based on their serum vitamin D levels, participants were categorized into three groups: those with severe deficiency (≤ 10 ng/ml), insufficiency (10–30 ng/ml), and sufficiency (≥ 30 ng/ml).

Participants in the severe vitamin D deficiency group (≤ 10 ng/ml) were prescribed 50,000 IU/week of vitamin D for 8 weeks, followed by 1,000 IU/day for 16 weeks as a maintenance dose. Adherence was monitored weekly via WhatsApp messages for the first 8 weeks [17]. Participants in the control group (vitamin D ≥ 30 ng/ml) did not receive supplementation. The control group (vitamin D ≥ 30 ng/ml) did not receive supplementation or a placebo and was only asked to return for repeat blood sampling after 6 months. At 6 months, all participants underwent repeat blood sampling for vitamin D, TC, HDL-C, Apo B and FRS reassessment.

### Statistical analysis

Continuous variables were presented as median (interquartile range), while categorical variables were expressed as numbers and percentages. To assess the normality of data distribution, the Kolmogorov-Smirnov test was performed. For group comparisons, Kruskal-Wallis and Mann-Whitney U tests were used to evaluate differences between vitamin D categories, while the Spearman correlation test was applied to examine correlations between vitamin D levels and other continuous variables. Additionally, Kendall’s Tau-b test was employed to analyze correlations between categorical variables.

To assess the impact of vitamin D correction, a Wilcoxon signed-rank test was conducted to compare measurements before and after the 6 months for the treatment and the control groups. All statistical analyses were performed using SPSS version 26, with a P-value < 0.05 considered statistically significant.

## Results

### Baseline characteristics

The current study included 201 participants, the median age of participants was 48 (34–63) years, and males represented 103 (51.2%) of participants. The median BMI was 31.6 (28–34) kg/m^2^. The median FRS was 4 (1–15). Males had a significantly higher age compared to females *p* = 0.001. Median FRS was significantly higher in males 13 (6–21) compared to females 1(1–4), *P* = 0.0001. Baseline characteristics of participants are explained in Table 1.

**Table 1:** Baseline characteristics of the participants

Variable	Total (*N* = 201)	Male (*N* = 103)	Female (*N* = 98)	*P*-value
Age (years)	48 (34–63)	51 (37–63)	41 (33–54)	0.001
BMI (kg/m²)	31.6 (28–34)	31.2 (27–34)	32 (28–35)	0.1
Systolic BP (mmHg)	124 (110–140)	125 (110–140)	124 (110–139)	0.94
Diastolic BP (mmHg)	77 (60–90)	79 (60–90)	74.5 (60–89)	0.07
Total Cholesterol (mg/dl)	199 (170–220)	200 (180–221)	190 (165–220)	0.1
Triglycerides (mg/dl)	158 (107–212)	189 (125–227)	138 (95–190)	0.0001
LDL-C (mg/dl)	120 (96–140)	125 (100–141)	118 (92–138)	0.1
VLDL-C (mg/dl)	31 (21–42)	37 (24–45)	27 (19–37)	0.0001
HDL-C (mg/dl)	43 (36–51)	41 (35–49)	45 (37–53)	0.006
Non-HDL-C (mg/dl)	153 (125–178)	159 (130–183)	147 (116–172)	0.03
Apo B (mg/dl)	130 (82–184)	148 (126–260)	87 (72–136)	0.0001
Vitamin D (ng/ml)	17 (10–32)	20 (10–36)	15 (10–29)	0.09
FRS	4 (1–15)	13 (6–21)	1 (1–4)	0.0001
Smoking (%)	100 (49.8%)	64 (62.1%)	36 (36.7%)	0.0001

Note: The Mann-Whitney U Test was used to compare the median difference between males and females. The chi-square test was used to compare the number and percentages of Numerical variables between males and females. N: number of participants, Continuous variables are measured as the median (IQ R). Numerical variables are measured as numbers and percentages. BMI: body mass index, Sys BP: systolic Blood pressure, Dias BP: diastolic blood pressure, LDL-C: low-density lipoprotein cholesterol, VLDL-C: very low-density lipoprotein cholesterol, HDL-C: high-density lipoprotein cholesterol, non-HDL-C: non-high-density lipoprotein cholesterol, Apo B: apolipoprotein B, FRS: Framingham Risk Score

Testing normality showed a non-normal distribution for the variables except BMI and HDL-C. Table 2 explains the pattern of age distribution according to vitamin D levels.

**Table 2:** Vitamin D levels and age distribution

Age groups	Vitamin D ≤ 10	Vitamin D 10–30	Vitamin D ≥ 30	Total
30–40 year	28(46.6%)	36(45.5%)	11(17.7%)	75
40.1–50 year	6(10%)	24(30.3%)	11(17.7%)	41
> 50 year	26(43.3%)	19(24%)	40 (64.5%)	85
Total N	60	79	62	201

Note: Kendall’s Tau-b = 0.2, *p* = 0.001

### Phase 1 Findings

A Comparison of the measured variables among the 3 groups of vitamin D as well as between every 2 groups of vitamin D was conducted and explained in Table 3 where a significant age difference, Apo B and FRS among the three groups of vitamin D was revealed.

**Table 3:** Comparison of the measured variables across vitamin D groups

Variables	Group 1: Vitamin D ≤ 10 ng/ml (*N* = 60)	Group 2: Vitamin D 10–30 ng/ml (*N* = 79)	Group 3: Vitamin D ≥ 30 ng/ml (*N* = 62)	(1, 2, 3) *P*-value	(1, 2) *P*-value	(1,3) *P*-value	(2,3) *P*-value
Age (years)	48 (32–59)	41 (33–50)	61 (46–66)	0.0001	0.4	0.0001	0.0001
BMI (kg/m²)	31 (26–35)	32 (28–35)	30 (28–34)	0.2	-	-	-
Vitamin D (ng/ml)	9 (8–10)	17 (13–23)	40 (32–49)	0.0001	0.0001	0.0001	0.0001
TC (mg/dl)	191 (159–210)	200 (170–231)	200 (178–238)	0.08	-	-	-
TG (mg/dl)	135 (84–191)	158 (106–220)	186 (129–231)	0.0001	0.008	0.0001	0.1
HDL-C (mg/dl)	39 (35–51)	43 (36–52)	44 (37–50)	0.5	-	-	-
LDL-C (mg/dl)	123 (91–138)	119 (99–141)	120 (99–138)	0.8	-	-	-
VLDL-C (mg/dl)	26 (16–34)	31 (21–44)	37 (25–46)	0.0001	-	0.0001	0.07
Non-HDL-C (mg/dl)	149 (110–165)	149 (110–165)	157 (126–185)	0.1	-	-	-
Apo B (mg/dl)	135 (72–185)	99 (78–145)	144 (115–261)	0.0001	0.3	0.02	0.0001
FRS	3.5 (1–17)	1 (1–6)	11.5 (4–18)	0.0001	0.06	0.01	0.0001

Note: Kruskal-Wallis H Test was used to compare the median differences between the three groups of vitamin D. Mann-Whitney U Test was used to evaluate the median differences between every two groups of vitamin D. 1 = severe vit D deficiency (≥ 10 ng/ml), 2 = vit D insufficiency (10–30 ng/ml), 3 = vit D sufficiency (D ≥ 30 ng/ml). Variables are measured as median (IQ R). N: number of participants, BMI: body mass index, TC: total cholesterol, TG: triglyceride, LDL-C: low-density lipoprotein cholesterol, VLDL-C: very low-density lipoprotein cholesterol, HDL-C: high-density lipoprotein cholesterol, non-HDL-C: non-high-density lipoprotein cholesterol, Apo B: apolipoprotein B

Group 1: Severe deficiency, Group 2: Insufficiency, Group 3: Sufficiency. P-values represent statistical comparisons across and between groups

**Fig. 2:**
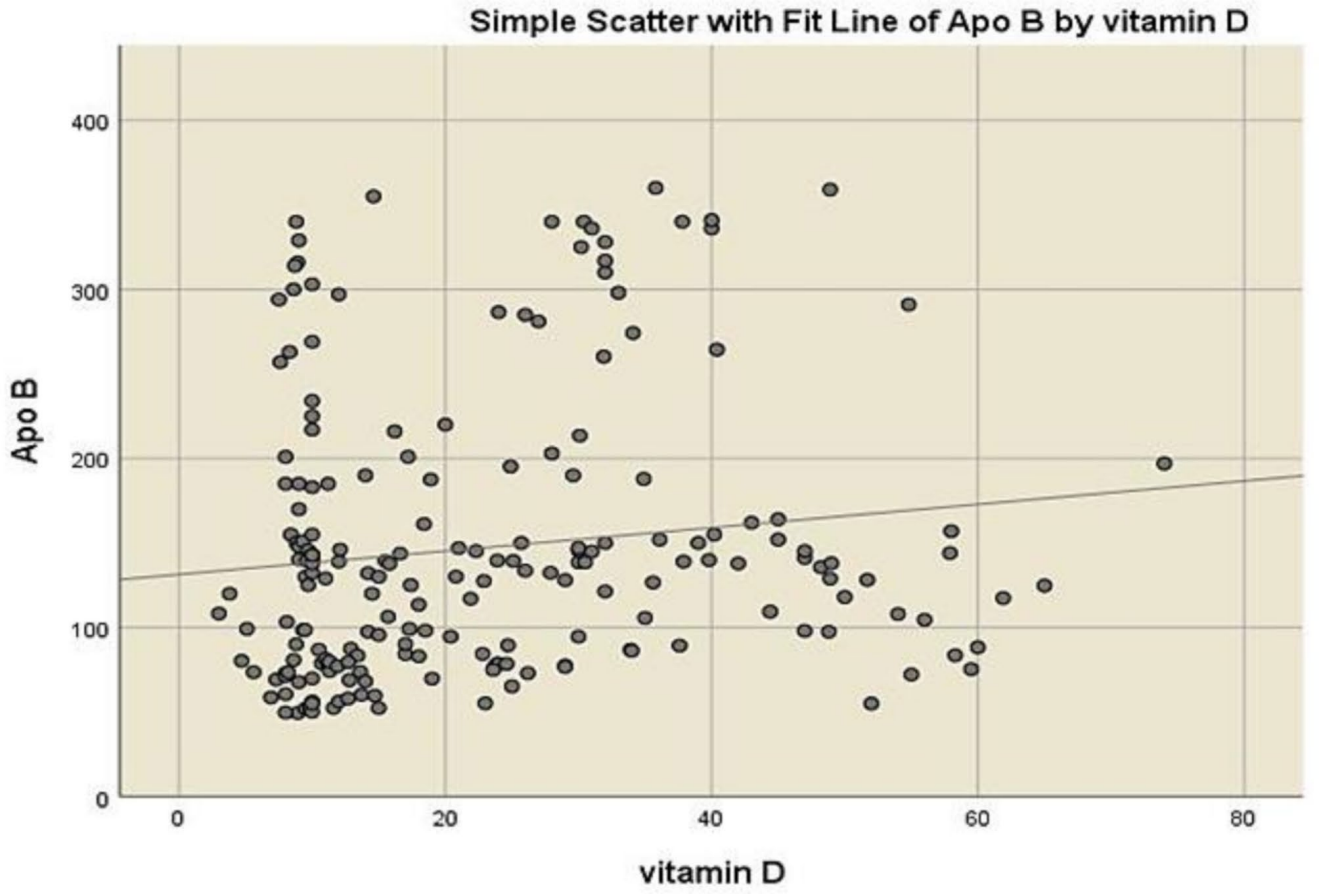
Scatter plot showing the correlation between serum Vitamin D and Apo B levels (*r* = 0.19, p-value = 0.007)

**Fig. 3:**
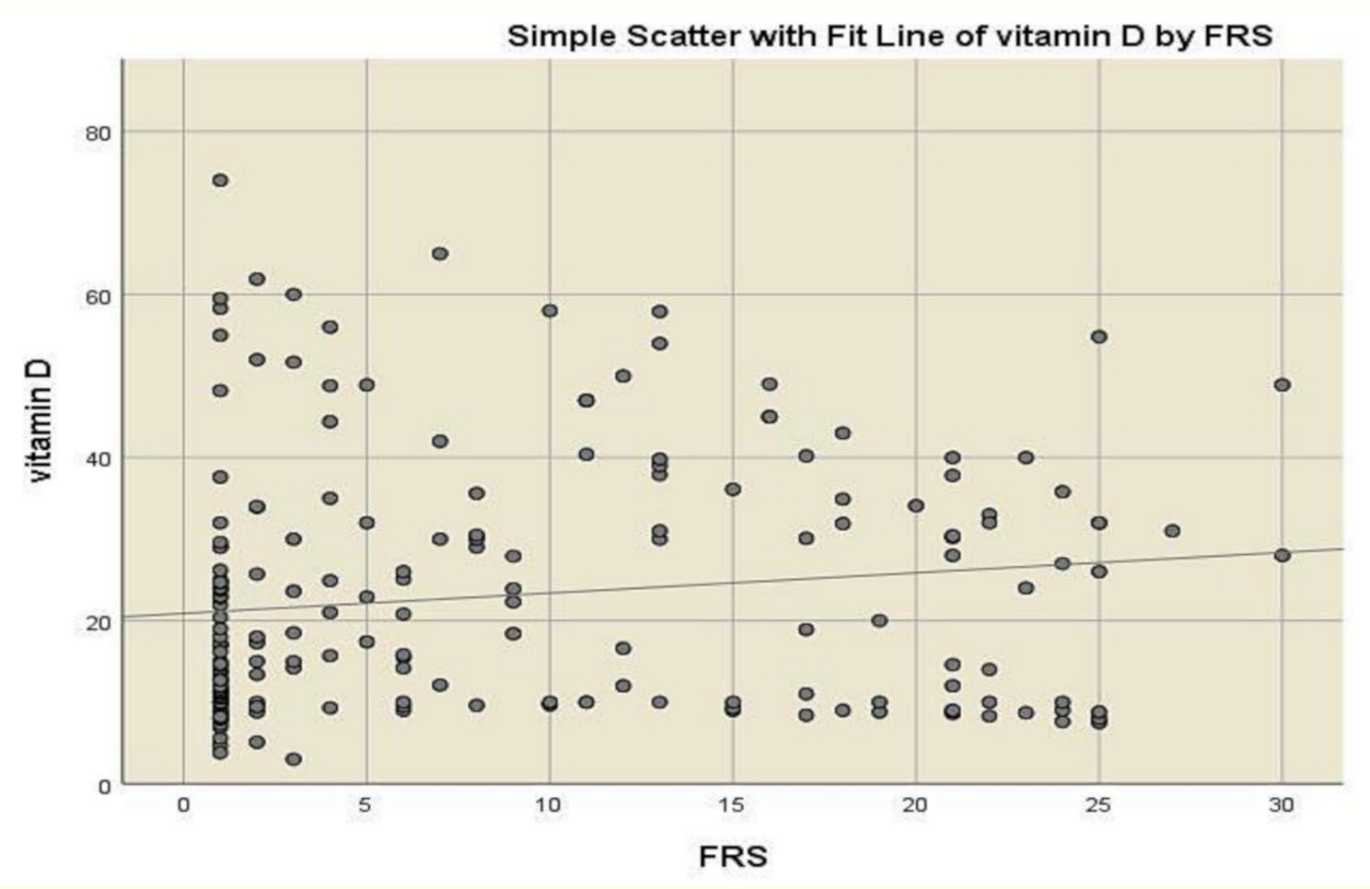
Scatter plot showing the correlation between serum Vitamin D and FRS (*r* = 0.2, p-value = 0.003)

The correlations between vitamin D and other variables are illustrated in Table 4 where a significant positive correlation between vitamin D and age, TC, Apo B and FRS was revealed. Spearman correlation analysis revealed a weak but significant positive correlation between vitamin D and Apo B (*r* = 0.19, *p* = 0.007), as well as between vitamin D and FRS (*r* = 0.2, *p* = 0.003). Table 4 presents correlation coefficients based on raw values, while visual representations in Figs. 2 and 3 include all individual data points, including outliers.

**Table 4:** Correlations of vitamin D with other variables (*N* = 201)

	Correlation with vitamin D	
Variables	r	*P*-value
Age	0.3	**0.0001**
BMI	0.01	0.8
TC (mg/dl)	0.1	0.01
TG (mg/dl)	0.28	0.0001
LDL-C (mg/dl)	0.04	0.5
VLDL-C (mg/dl)	0.3	0.0001
HDL-C (mg/dl)	0.06	0.3
non-HDL-C (mg/dl)	0.1	0.04
FRS	0.2	0.003
Apo B	0.19	0.007

Note: Correlation is significant at p-value < 0.05 level (2-tailed). Spearman Rank-Order Test was used to analyze correlation. r: correlation coefficient. Apo B: Apolipoprotein B, BMI: body mass index, TC: total cholesterol, TG: triglyceride, VLDL-C: very low-density lipoprotein, non-HDL-C: non-high-density lipoprotein cholesterol, LDL-C: low-density lipoprotein cholesterol, HDL-C: high-density lipoprotein cholesterol, FRS: Framingham Risk Score

### Phase 2 Findings

**Fig. 4:**
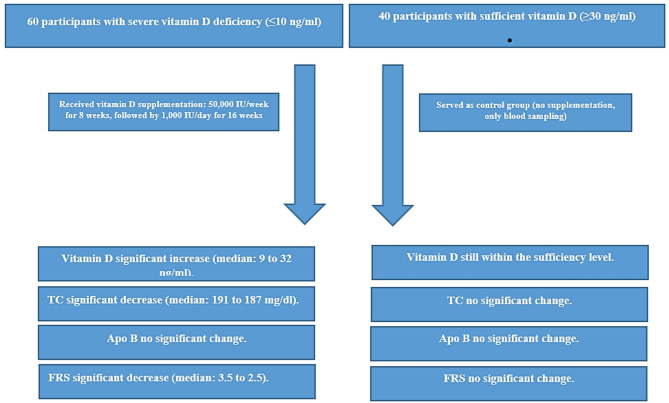
Flowchart of participant progression and key outcomes after 6 months of Vitamin D correction

A comparison of the changes in the measured variables before and after vitamin D correction in the treatment group, and of the changes in the measured variables at the baseline and the end of 6 months in the control groups is shown in Table 5. After vitamin D supplementation, the 60 participants in the treatment group experienced a significant increase in serum level of vitamin D and a significant decrease in serum level of TC (*p* = 0.004) and FRS (*p* = 0.007), and no significant changes in Apo B. Of the 40 participants in the control group, both HDL-C and vitamin D were significantly decreased. Nevertheless, vitamin D remained within the sufficiency level. Figure 4 demonstrate the Key Outcomes After 6 Months of Vitamin D Correction.

**Table 5:** Differences of the measured variables before and after vitamin D correction for the treatment group and at the baseline and the end of 6 months for the control group

Variables	Treatment group (*N* = 60)			Control group (*N* = 40)		
	(Before vit D supplementation) Median (IQ *R*) *N* = 60	(After vit D supplementation) Median (IQ *R*) *N* = 60	*p*-value	(1st measurement) Median (IQ *R*) *N* = 40	(2nd Median measurement) (IQ *R*) *N* = 40	*p*-value
Age	48(30–75)			63(30–77)		
Vit D (ng/ml)	9 (8–10)	32 (26–36)	0.0001	39.5(32–48)	38 (32–44)	0.001
Sys BP(mmHg)	127(111–139)	125(110–140)	0.3	123(110–140)	122(111–140)	0.2
TC (mg/dl)	191 (159–210)	187 (167–202)	0.004	197 (171–217)	199 (167–214)	0.1
TG (mg/dl)	135 (84–191)	128 (81–192)	0.054	175 (124–211)	165 (119–165)	0.1
VLDL-C (mg/dl)	27 (16–36)	25 (16–34)	0.05	35(24–42)	33 (23–42)	0.1
LDL-C (mg/dl)	123 (91–138)	116 (95–134)	0.051	115 (85–138)	119 (74–136)	0.4
HDL-(mg/dl)	39 (35–51)	40 (37–49)	0.5	46 (39–51)	42 (39–49)	0.01
Non-HDL-C (mg/dl)	149 (110–165)	147 (110–159)	0.004	154 (116–173)	149 (121–170)	0.5
Apo B	135 (72–185)	119(76–186)	0.2	150(118–302)	149 (116–265)	0.9
FRS	3.5 (1–17)	2.5(1–17)	0.007	13 (4–20)	13 (5–19)	0.7

Note: The Willcoxon Signed Rank Test was used to compare the alteration in the median (IQ R) in the treatment group (60 participants) and the control group (40 participants) separately before and after the course of vitamin D correction for those in the treatment group. Sys BP: systolic BP, TC: total cholesterol, TG: triglyceride, VLDL-C: very low-density lipoprotein cholesterol. Apo B: Apolipoprotein B, non-HDL-C: non-high-density lipoprotein cholesterol, LDL-C: low-density lipoprotein cholesterol, HDL-C: high-density lipoprotein cholesterol. FRS: Framingham risk score

Sex variation in response to vitamin D correction was noticeable in phase 2 as shown in Table 6 Males have demonstrated a significant decrease in FRS and TC as well as a significant increase in HDL-C and no significant changes in Apo B. However, females have demonstrated no parallel results.

**Table 6:** Sex-Based differences in the impact of vitamin D supplementation on lipid profile, Apo B, and FRS

	Females *N* = 30			Male *N* = 30		
	**(Before vit D supplementation)**	**(After vit D supplementation)**	*P* **-value**	**(Before vit D supplementation)**	**(After vit D supplementation)**	*P* **-value**
Age	34 (30–52)			55 (33–66)		
Vit D	9 (8–10)	30 (23–36)	0.0001	9 (8-9.7)	34 (29–37)	0.0001
TC	175 (155–202)	186 (156–200)	0.09	200 (170–210)	188 (175–205)	0.02
TG	96 (76–140)	103 (72–152)	0.7	175 (108–207)	167 (103–203)	0.02
HDL-C	45 (37–57)	41 (35–52)	0.3	37 (34–45)	40 (37–46)	0.04
VLDL-V	19 (15–28)	20 (14–29)	0.7	35 (19–41)	33 (19–40)	0.02
LDL-C	110 (87–135)	115 (80–142)	0.3	127 (102–139)	116 (105–129)	0.05
Non-HDL-C	132 (105–157)	141 (102–157)	0.1	159 (130–175)	148 (123–169)	0.007
FRS	1 (1-2.5)	1 (1–3)	0.4	15 (6.7–22)	14 (2–20)	0.01
Apo B	76 (58–140)	81 (67–130)	0.9	150 (123–258)	142 (115–285)	0.09

Note: The Willcoxon Signed Rank Test was used to compare the alteration in the median (IQ R) before and after the vitamin D correction course for the 30 female participants and 30 male participants who completed phase 2 of the study. TC: total cholesterol, TG: triglyceride, Apo B: Apolipoprotein B, non-HDL-C: non-high-density lipoprotein cholesterol, LDL-C: low-density lipoprotein cholesterol, VLDL-C: very low-density lipoprotein cholesterol, HDL-C: high-density lipoprotein cholesterol. FRS: Framingham risk score

## Discussion

The findings from this study provide valuable insights into the association between serum vitamin D with lipid, Apo B and cardiovascular risk, particularly within an understudied Iraqi population. In the cross-sectional phase, a significant positive correlation between serum vitamin D and age was observed (P value = 0.001).

An Iraqi study conducted by Amen et al. (2020) [18] found a significantly higher prevalence of severe vitamin D deficiency among patients with acute myocardial infarction compared to healthy controls, suggesting that vitamin D deficiency may contribute to cardiovascular pathology through mechanisms such as endothelial dysfunction and increased inflammatory response. Another Iraqi study conducted by Al-Tu’ma et al. (2017) [19] demonstrated a significant correlation between low vitamin D levels and unstable angina, emphasizing its role in lipid metabolism and atherogenic balance, particularly through its association with the Apo B/Apo A1 ratio, a key marker of cardiovascular risk. The finding appears to be influenced by confounding impact of age distribution of participants with serum levels of vitamin D, as a larger proportion of older individuals had sufficient vitamin D levels (> 50 years: 64.5%), whereas nearly half of those with severe deficiency (46.6%) were younger (30–40 years). Despite their increased outdoor activity, younger individuals may exhibit lower compliance with routine medical screenings, leading to undiagnosed vitamin D deficiency. However, the observed higher vitamin D levels in older subjects compared to younger individuals may be attributed to increased routine medical screenings and higher vitamin D supplementation among the elderly population. This positive correlation aligns with previous studies in Saudi Arabia and Iraq [17, 20, 21], reinforcing the notion that aging does not necessarily lead to lower vitamin D levels. Interestingly, an Iranian study further supports these findings, suggesting that vitamin D levels may remain stable with age, contradicting the expected physiological decline due to reduced skin synthesis, decreased renal activation, and intestinal resistance [22]. The current study also found higher median vitamin D levels in males compared to females, consistent with findings from Saudi Arabia and Iraq [23, 24]. This discrepancy can largely be attributed to sociocultural behaviours, where women are more likely to experience reduced sun exposure due to conservative dress styles.

In phase 1, the significant difference of FRS among the 3 groups of vitamin D reflects the impact of age distribution, group 3 had the higher age, which contributed to their elevated FRS as age is a major determinant of both TC levels and FRS values, and is also closely linked to hypertension, which was present but medically controlled in our cohort and did not show significant variation post-intervention. In the prospective phase, one of the most remarkable findings was that correcting severe vitamin D deficiency led to a significant decrease in TC levels, which was accompanied by a corresponding decrease in FRS (P value = 0.004). The control group, in contrast, exhibited no significant changes. These findings suggest that vitamin D supplementation may influence lipid metabolism, potentially lowering cardiovascular disease risk. The proposed mechanisms linking vitamin D to lipid metabolism include its role in increasing hepatic calcium concentrations, which may reduce triglyceride secretion. Additionally, vitamin D has been shown to stimulate apolipoprotein A1 (apoA1) expression, which enhances HDL-C synthesis and promotes reverse cholesterol transport, ultimately leading to a decrease in circulating TC levels [25]. These results align with previous meta-analyses [26], which also reported lipid-lowering effects following vitamin D correction. A study in pregnant women with gestational diabetes reported a decrease in TC after two doses of 50,000 IU vitamin D, administered 21 days apart, at baseline serum levels of 20.44 ng/mL [27]. Similarly, among HIV patients with baseline vitamin D ≤ 20 ng/mL, daily supplementation of 4,000 IU for 12 weeks resulted in a significant TC reduction [28]. In Saudi patients with type 2 diabetes, vitamin D supplementation also led to significant reductions in both TC and LDL-C levels [29]. Interestingly, a study on atorvastatin-treated patients found that therapeutic doses of vitamin D significantly decreased TC and LDL-C levels, despite lowering atorvastatin concentrations [4]. These findings suggest that vitamin D may exert an independent lipid-lowering effect, similar to the additional cholesterol reduction achieved with ezetimibe or increased statin dosage. However, other studies have reported no significant changes in TC following vitamin D supplementation, while some have even observed increases in TC levels, highlighting the ongoing debate regarding vitamin D’s role in lipid metabolism [30, 31].

This study examined the association between serum vitamin D and Apo B and the impact of vitamin D correction on Apo B levels. In the cross-sectional phase, a weak but significant positive correlation was observed between vitamin D and Apo B (*r* = 0.18, *p* = 0.007), likely influenced by age distribution, as younger participants tended to have lower vitamin D levels, whereas older individuals, who often receive supplementation, had higher levels. However, few studies have addressed the relationship between vitamin D and Apo B. Notably, a Japanese study involving 136 healthy males reported a significant positive correlation between serum vitamin D and Apo B, despite strict exclusion of confounding factors such as diabetes, cardiovascular disease, and lipid-lowering therapy [32].

A recent meta-analysis of randomized controlled trials on vitamin D supplementation examined its effects on Apo A and Apo B levels across eight interventional studies [8]. Among these, three reported no significant change in Apo B, three observed a significant decrease, and one noted an increase [33]. These conflicting results highlight ongoing uncertainties regarding the physiological impact of vitamin D on Apo B metabolism and underscore the need for further investigation to clarify the underlying mechanisms.

However, in the prospective phase, despite a significant decrease in TC following vitamin D supplementation, Apo B levels remained unchanged. This suggests that Apo B metabolism is not directly affected by short-term vitamin D correction. A potential explanation is that the significant decrease in TC was not substantial enough to influence Apo B synthesis, or that Apo B requires a longer time to exhibit measurable changes after vitamin D correction.

The association between vitamin D and cardiovascular disease remains controversial. While several studies report no significant benefit of vitamin D supplementation on cardiovascular disease outcomes [10, 34, 35], the current study demonstrates a clear decrease in cardiovascular risk, as estimated by FRS, following vitamin D correction. This significant decrease appears to be largely mediated by the decrease in TC levels, further reinforcing vitamin D’s potential role in cardiovascular risk modification. To date, limited numbers of studies have specifically examined the association between vitamin D and FRS. One study in overweight or obese postmenopausal women found no significant association, possibly due to a small sample size or low baseline FRS in the population [36]. Additionally, two studies from Turkey [37] and Korea [38] reported a negative correlation between vitamin D levels and cardiovascular risk scores.

These findings reinforce the role of vitamin D deficiency as a cardiovascular risk factor. A similar study in Saudi Arabia assessed the impact of vitamin D correction on the 10-year atherosclerotic cardiovascular disease risk score and reported comparable findings [39]. The results suggested that vitamin D supplementation significantly lowered cardiovascular disease risk, largely attributed to increases in HDL-C levels. Notably, both studies observed sex-based differences. The current study demonstrated that males experienced a significant decrease in TC and cardiovascular risk scores compared to females, attributing this to the older age and higher baseline TC levels in male participants, making them more responsive to intervention.

Similarly, a study by Choi et al. (2015) [40] assessed dietary calcium intake and its correlation with cardiovascular risk using the FRS. The study found that individuals with both low and excessive calcium intakes exhibited higher cardiovascular risk, particularly in vitamin D-deficient males, highlighting the complex interaction between vitamin D status, calcium metabolism, and cardiovascular health.

One of the major strengths of the current study is the adequate follow-up period, allowing for a steady-state concentration of 25-hydroxyvitamin D to be achieved following supplementation. The study also exclusively targeted individuals with severe vitamin D deficiency, unlike previous studies that included participants with insufficient or normal vitamin D levels. Additionally, some prior studies permitted control participants to continue minimal daily vitamin D supplementation (600–800 IU/day), which could have influenced their results [35]. Despite these strengths, some limitations must be acknowledged. The age distribution of participants may have influenced findings, particularly in the cross-sectional analysis. Furthermore, the control group did not include individuals with severe vitamin D deficiency, as it would have been ethically unacceptable to leave them untreated for six months. Finally, the FRS was originally developed for Western populations, and its applicability to an Iraqi cohort warrants further validation.

This variability in the effect of vitamin D on Apo B and FRS is also reflected in other cardiovascular biomarker studies. For instance, although the Framingham Heart Study linked ABO blood groups to CAD risk, a more recent study found no association between ABO blood group and thrombus burden in STEMI patients, suggesting that the predictive value of such markers may be population- or context-dependent [41].

Although our study did not evaluate the direct predictive value of Apo B and FRS for cardiovascular outcomes, the observed changes in FRS following vitamin D correction suggest that variations in vitamin D status may influence cardiovascular risk estimation. Particularly, individuals with severe deficiency exhibited elevated FRS, which decreased significantly upon correction.

Given the high prevalence of vitamin D deficiency in Iraq [42] and its observed association with elevated FRS, early detection and correction of severe deficiency could represent a cost-effective preventive strategy for reducing cardiovascular risk. Integrating vitamin D screening into routine clinical evaluations may not only optimize cardiovascular risk stratification but also alleviate the broader public health burden of CAD management in this population. This study provides compelling evidence that correcting severe vitamin D deficiency leads to a significant decrease in TC levels and cardiovascular risk, as estimated by FRS. Future research should focus on developing region-specific cardiovascular risk assessment tools and further exploring vitamin D’s mechanistic effects on lipid metabolism and cardiovascular disease progression.

## Conclusion

Correction of severe vitamin D deficiency was associated with a significant reduction in TC and cardiovascular risk, particularly in males as estimated by the FRS in Phase 2 of the study. However, Apo B levels remained unchanged after vitamin D correction, suggesting that vitamin D may not directly influence Apo B metabolism in the short term. The observed weak but significant positive correlation between vitamin D and Apo B in the cross-sectional phase may have been influenced by the age distribution of participants, highlighting the need for further investigation. These findings suggest that severe vitamin D deficiency should be corrected prior to cardiovascular risk estimation, as vitamin D may act as a modifiable factor influencing lipid metabolism and FRS. Future research should explore long-term effects, potential sex-based differences, and vitamin D’s broader role in lipid metabolism beyond Apo B regulation.

## Electronic supplementary material

Below is the link to the electronic supplementary material.

Supplementary Material 1

Supplementary Material 2

Supplementary Material 3

Supplementary Material 4

## Data Availability

The dataset supporting the findings of this study is currently restricted access and available upon request. It is hosted on Zenodo under the DOI 10.5281/zenodo.14918527. Upon acceptance of this manuscript for publication, the dataset will be made publicly available on Zenodo, ensuring open access to all relevant data used in this study. For citation purposes, please refer to: Nathir Ahmed, I. (2025). Serum Apolipoprotein B [Data set]. Zenodo. DOI: 10.5281/zenodo.14918527.

## Declarations

### Clinical trial number

The study has been successfully retrospectively registered and approved by the Thai Clinical Trials Registry (TCTR) under the identification number TCTR20250301003 on the 1st of March 2025. This registration affirms the study’s adherence to ethical and methodological standards in clinical research.

## Abbreviations

Apo B: Apolipoprotein B
BMI: Body Mass Index
BP: Blood Pressure
CAD: Coronary Artery Disease
ELISA: Enzyme–Linked Immunosorbent Assay
FRS: Framingham Risk Score
HDL: C–High–Density Lipoprotein Cholesterol
IU: International Units
LDL: C–Low–Density Lipoprotein Cholesterol
RCT: Randomized Controlled Trial
SPSS: Statistical Package for the Social Sciences
STROBE: Strengthening the Reporting of Observational Studies in Epidemiology
TC: Total Cholesterol
TG: Triglycerides
USM: Universiti Sains Malaysia
VLDL: C–Very Low–Density Lipoprotein Cholesterol
