# Correction: The correlation between serum vitamin D with Apo B and framingham risk score among a group of Iraqi subjects: a cross-sectional and prospective pilot study

**DOI:** 10.1186/s12872-025-05117-5

**Published:** 2025-08-21

**Authors:** Israa Nather Ahmed, Fatimatuzzahra’ Abd Aziz, Raid Dhia Hashim

**Affiliations:** 1 Discipline of Clinical Pharmacy, School of Pharmaceutical Sciences, Universiti Sains Malaysia, Pulau Pinang, Malaysia; 2 Pharmacy Department, Al Rasheed University College, Baghdad, Iraq; 3 Discipline of Clinical Pharmacy, School of Pharmaceutical Sciences, Universiti Sains Malaysia, Pulau Pinang, 11800 Malaysia; 4 College of Pharmacy, Al-Farahidi University, Baghdad, Iraq

**Correction: BMC Cardiovascular Disorders 25, 445 (2025)**

**https://doi.org/10.1186/s12872-025-04855-w**

In this article [1], the author’s name Fatimatuzzahra’ Abd Aziz was incorrectly written as Fatimatuzzahra’ Abd Aziz Binti.

The affiliation details for Author Fatimatuzzahra’ Abd Aziz were incorrectly given as ‘College of Pharmacy, Al-Farahidi University, Baghdad, Iraq’ but should have been ‘Discipline of Clinical Pharmacy, School of Pharmaceutical Sciences, Universiti Sains Malaysia, Pulau Pinang, Malaysia’.

The affiliation details for Author Raid Dhia Hashim were incorrectly given as‘Discipline of Clinical Pharmacy, School of Pharmaceutical Sciences, Universiti Sains Malaysia, Pulau Pinang, Malaysia’ but should have been ‘College of Pharmacy, Al-Farahidi University, Baghdad, Iraq’.

The original article has been corrected.
