# Supplementary figures and images for: The correlation between serum vitamin D with Apo B and framingham risk score among a group of Iraqi subjects: a Cross-sectional and prospective pilot study
Source: BMC Cardiovasc Disord. 2025 Jul 3;25:445. doi: 10.1186/s12872-025-04855-w (PMC12224530; doi:10.1186/s12872-025-04855-w)

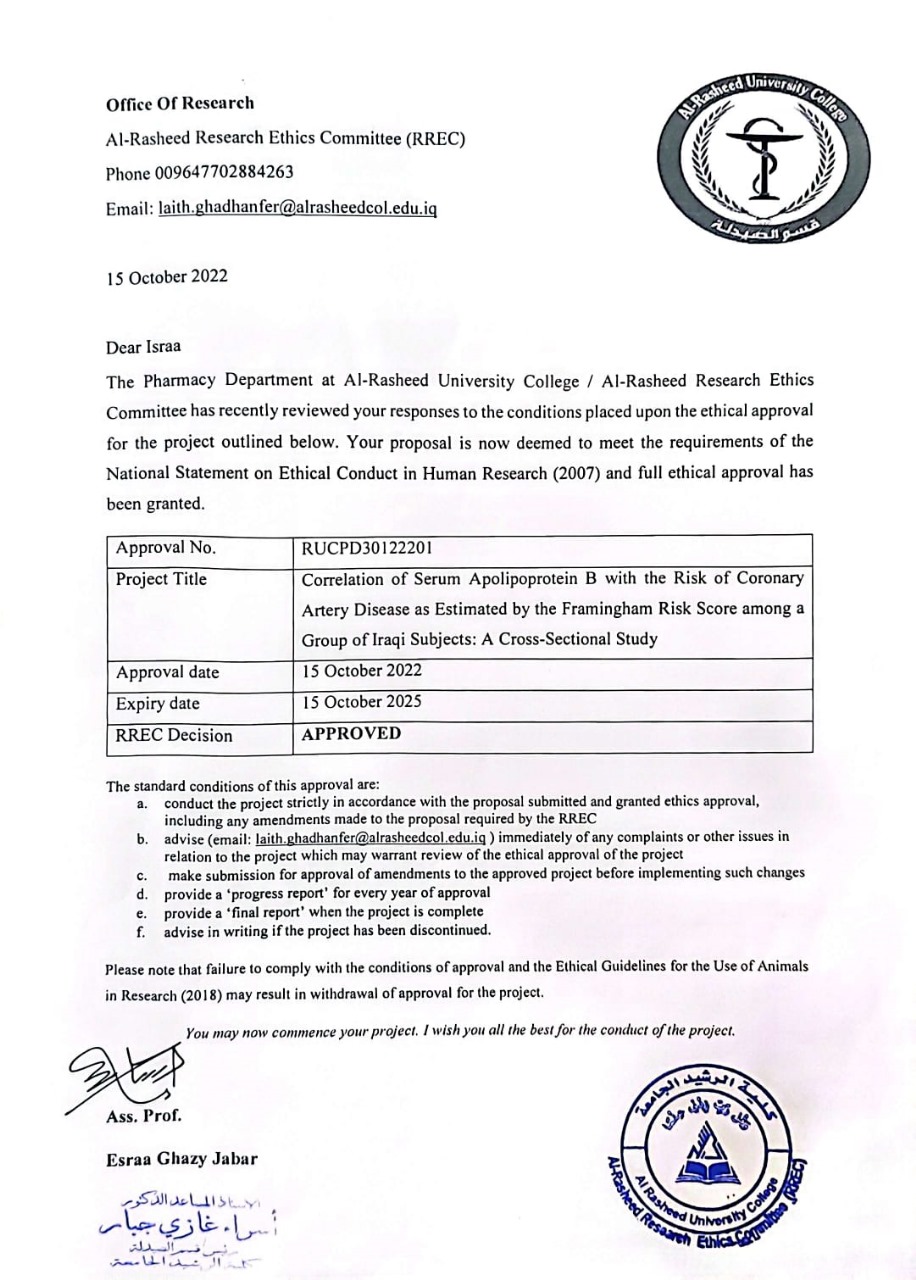

Supplement: Supplementary file 1 — Supplementary Material 1 [file 12872_2025_4855_MOESM1_ESM.jpg]
