# Supplementary material for: The correlation between serum vitamin D with Apo B and framingham risk score among a group of Iraqi subjects: a Cross-sectional and prospective pilot study
Source: BMC Cardiovasc Disord. 2025 Jul 3;25:445. doi: 10.1186/s12872-025-04855-w (PMC12224530; doi:10.1186/s12872-025-04855-w)
Supplement: Supplementary file 2 — Supplementary Material 2 [file 12872_2025_4855_MOESM2_ESM.pdf]

Rania Al-taie

Poor Ovarian Reserve in Young Infertile Women

Quick Submit

Quick Submit

IBAZ

Document Details

Submission ID

trn:oid:::1:3157026417

Submission Date

Feb 16, 2025, 11:56 PM GMT+3

Download Date

Feb 17, 2025, 12:00 AM GMT+3

File Name

Version_3_POR_forplagarism_check.docx

File Size

64.4 KB

8 Pages

2,384 Words

14,488 Characters

0% detected as AI

The percentage indicates the combined amount of likely AI-generated text as well as likely AI-generated text that was also likely AI-paraphrased.

Caution: Review required.

It is essential to understand the limitations of AI detection before making decisions about a student's work. We encourage you to learn more about Turnitin's AI detection capabilities before using the tool.

Detection Groups

1 AI-generated only 0%
Likely AI-generated text from a large-language model.

2 AI-generated text that was AI-paraphrased 0%
Likely AI-generated text that was likely revised using an AI-paraphrase tool or word spinner.

Disclaimer
Our AI writing assessment is designed to help educators identify text that might be prepared by a generative AI tool. Our AI writing assessment may not always be accurate (it may misidentify writing that is likely AI generated as AI generated and AI paraphrased or likely AI generated and AI paraphrased writing as only AI generated) so it should not be used as the sole basis for adverse actions against a student. It takes further scrutiny and human judgment in conjunction with an organization's application of its specific academic policies to determine whether any academic misconduct has occurred.

Frequently Asked Questions

How should I interpret Turnitin's AI writing percentage and false positives?
The percentage shown in the AI writing report is the amount of qualifying text within the submission that Turnitin's AI writing detection model determines was either likely AI-generated text from a large-language model or likely AI-generated text that was likely revised using an AI-paraphrase tool or word spinner.

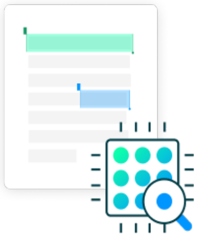

False positives (incorrectly flagging human-written text as AI-generated) are a possibility in AI models.

AI detection scores under 20%, which we do not surface in new reports, have a higher likelihood of false positives. To reduce the likelihood of misinterpretation, no score or highlights are attributed and are indicated with an asterisk in the report (*%).

The AI writing percentage should not be the sole basis to determine whether misconduct has occurred. The reviewer/instructor should use the percentage as a means to start a formative conversation with their student and/or use it to examine the submitted assignment in accordance with their school's policies.

What does 'qualifying text' mean?
Our model only processes qualifying text in the form of long-form writing. Long-form writing means individual sentences contained in paragraphs that make up a longer piece of written work, such as an essay, a dissertation, or an article, etc. Qualifying text that has been determined to be likely AI-generated will be highlighted in cyan in the submission, and likely AI-generated and then likely AI-paraphrased will be highlighted purple.

Non-qualifying text, such as bullet points, annotated bibliographies, etc., will not be processed and can create disparity between the submission highlights and the percentage shown.

Poor Ovarian Reserve in Young Infertile Women: Narrative Review

Abstract

Background: Poor ovarian reserve (POR) means a decrease in the quantity and quality of oocytes with subsequent decline of reproductive potential and/or insufficient response to ovulation induction.

Aim: This narrative review aimed to summarize causes/risks of decreased ovarian reserve and to highlight for the in-vitro fertilization (IVF) specialists to screen women with poor response to ovulation induction for POR and its causes.

Methods: Google and PubMed search was done to retrieve the definition, causes, diagnosis and pathophysiology of POR.

Results: About 20% of fragile X-associated primary ovarian insufficiency (FXPOI) syndrome had POR. Single-nucleotide polymorphisms (SNPs) of FSH receptors is commonly associated with POR. Covid-19 targeting the gonadotropin expression in the female reproductive system. HIV could reduce the ovarian reserve through oxidative stress manifested by ROMO1 (mitochondrial membrane protein). Chemotherapy breaks the double stranded DNA which leads to direct primordial follicle injury. All hemostatic methods used during surgical removal of endometriotic cysts, resulted in diminished ovarian reserve. Systemic lupus erythematosus (SLE) women of reproductive age have lower ovarian reserve compared to normal controls. Ovarian follicles may be damaged directly by hyperglycemia and/or indirectly by the oxidative of DM. Wi-Fi waves could have direct adverse effects on ovarian granulosa cells with subsequent abnormal folliculogenesis. Obese women during IVF programs are at risks of higher ovulation induction doses, fewer retrieved oocytes and higher failure rate. AMH is low in obese women compared to non-obese controls. Endocrine-disrupting chemicals (EDCs) can disrupt the ovarian hormonal function with subsequent anovulation, infertility and premature ovarian failure (POF).

Conclusion: This review identified new causes/risks of POR (i.e., Wi-Fi waves and EDCs). The effect of these new causes/risks on the ovarian reserve needs future studies and needs an effective preventive strategy.

Keywords: Anti-Mullerian hormone, Antral follicle count, Poor ovarian reserve.

Poor Ovarian Reserve in Young Infertile Women: Narrative Review

Introduction

Ovarian reserve is an essential determinant of female fertility, which normally decline, after 35 years of age. Poor ovarian reserve (POR) means a decrease in the quantity and quality of oocytes with subsequent decline of reproductive potential and/or insufficient response to ovulation induction. POR may occur due to diverse etiological/risk factors including genetic, environmental, infection, iatrogenic, and autoimmune disorders [1].

The female oocytes reach six to seven million during fetal life, then decline to 1 to 2 million oocytes at birth. At puberty the ovary contains 300-400 thousand oocytes and only one oocyte grows to dominant follicle from several growing follicles every month. At reproductive age, every woman loss 400-500 oocyte with each menstrual cycle and the ovary contains around 10 thousand oocytes by the age of 40 years [2].

About 9-24% of women subject to ovulation induction during in-vitro fertilization (IVF) programs have POR and poor reproductive outcome [3].

Bologna criteria (BC) decrease the discrepancy underlying POR definition [4]. The Bologna criteria define POR with at least two of the subsequent findings; A) Maternal age above or equal 40. B) Less than or equal ≤3 oocytes retrieved after standard stimulation protocol (poor response) and an abnormal ovarian reserve test (ORT).

A young woman can be diagnosed with POR if she showed a poor ovarian response twice for the maximal ovarian stimulation protocol or after abnormal ORT [4].

ORT includes; basal serum FSH and E_2 (i.e., elevated day 2-day 4 serum FSH is specific but not sensitive for diagnosing POR). Serum E_2 is not a significant tool to identify POR but the serum E_2 tends to be elevated (>60-80 pg/mL) in women with POR. Clomiphene citrate challenge test is not currently used for diagnosing POR.

Anti-Müllerian hormone (AMH) considered a helpful sensitive diagnostic tool as it remains constant within and between menstrual cycles. AMH is produced by granulosa cells of early growing follicles (normal AMH 1-4 ng/mL) [5].

Antral follicle count (AFC) means numbers of the ovarian follicles detected within the ovaries during early follicular phase and measuring 2-10 mm in size using the transvaginal sonography (TVS) (normal AFC 5-10 and it is operator dependent) [6].

It is important to differentiate between POR and premature ovarian failure (POF). POF occurs in women≤ 40 years before the typical age of menopause. POF typically presents with amenorrhea >6 months, after complete depletion of ovarian follicles and complete loss of ovarian hormonal function [7].

This narrative review aimed to summarize causes/risks of decreased ovarian reserve and to highlight for the in-vitro fertilization (IVF) specialists to screen women with poor response to ovulation induction for POR and its causes.

Methods: Google and PubMed search was done to retrieve the definition, causes, diagnosis and pathophysiology of POR.

Discussion

POR may occur due to diverse etiological factors including genetic, environmental, infection, iatrogenic, and autoimmune disorders but in most cases, no identifiable cause could be found. **Table 1** shows the causes of POR.

Genetic factors: It is recommended to screen young woman with POR for the possibility of genetic abnormalities [8].

FMR1 gene (Fragile X Messenger Ribonucleoprotein-1) is a protein coding gene, and it is linked to reduced ovarian reserve and neuropsychiatric disorder. About 20% of fragile X-associated primary ovarian insufficiency (FXPOI) syndrome had POR [9].

Single-nucleotide polymorphisms (SNPs) is another gene located in the human genome, provoked by point mutation and encoding many hormone receptors that are important in female fertility like AMH (AMHR2), FSH (FSHR), LH (LHR), estrogen (ESR), growth and differentiation factors (GDF9). SNPs of FSH receptors is commonly associated with POR [10].

FSHR starts a signaling cascade in the granulosa cells, responsible for ovarian follicles maturation, where its inactivation leads to the arrest of ovarian follicles maturation with subsequent anovulation and reduced ovarian reserve. SNPs 29G>A and 919G>A in FSHR may be associated with DOR [11].

Environmental factors: Environmental pollutants, and smoking accelerate ovarian follicles atresia, and deteriorate ovarian reserve quickly [12].

Epidemics: Like COVID-19 has high affinity to ACE2 receptors, which is widely expressed through the human body in pneumocytes, gastrointestinal, urinary, cardiovascular and nervous systems [13].

COVID-19 targeting the gonadotropin expression in the female reproductive system (i.e., ACE2 dependent) [14,15].

Clinical studies have been conducted to investigate the potential impact of COVID-19 vaccines on women's reproductive health, specifically their ovarian reserve with no conclusive results till now [16,17]. Scientists explained this by the limited number of cases and still they need time to evaluate the long-term side effect of Covid-19 vaccine on ovarian reserve [18,19].

Genital infections

1. Female genital tuberculosis (TB): The steroids-rich ovarian environment contributes to both genital tuberculosis and latency. Genital tuberculosis reaches ovaries through hematogenous route and can reduce ovarian reserve by many mechanisms including atrophic endometrium and adhesions.

Genital tuberculosis can result in considerable morbidities including ectopic pregnancy, menstrual dysfunction, and tubo-ovarain mass [20]. Genital tuberculosis involves mainly fallopian tubes (90%), endometrium (50-80%), ovaries (20-30%), and cervix (5–10%). It is important to diagnose TB early to prevent its major consequences (i.e., uterine synechiae, pelvic adhesions, and Fitz–Hugh–Curtis syndrome) [21]. Late TB diagnosis is associated with diminished ovarian reserve, especially with advanced maternal age.

2. Human Papilloma virus (HPV): Is the most prevalent sexually transmissible infection. HPV high-risk subtypes responsible for cancer cervix. HPV causes cancer through disruption of cell cycle regulation and genetic damage of infected cells.

HPV could lead to ovarian dysfunction and diminished ovarian reserve through its direct effect on ovaries and/or through persistent HPV and chronic inflammatory process [22].

3. Human immunodeficiency virus (HIV): Is a sexually transmitted disease and women with HIV are at risk of infertility than others. HIV could reduce the ovarian reserve and AMH through oxidative stress and oxygen radicals manifested by ROMO1 (mitochondrial membrane protein) [23]. Moreover, Tumor necrosis factor (TNF) influences serum AMH levels through modulation of granulosa cell function [23].

Chronic inflammatory disorders: Like Crohn's disease is a chronic inflammatory disease that causes irreversible damage to gastrointestinal tract. Active and advanced Crohn's could alter AMH level, cause infertility, and decreased ovarian reserve [24-26].

Iatrogenic factors

1. Drug-induced ototoxicity: Chemotherapy could affect the women's ovaries directly and/or indirectly [27].

Ovarian reserve diminishes after chemotherapy. The fertility returns to normal 6 months after chemotherapy, however this declined ovarian reserve following chemotherapy shortens the fertility time and results in POF years after chemotherapy [28].

The indirect effect of chemotherapy on the ovaries is expressed through upregulation in the PI3K signaling pathway which results in over phosphorylation of important proteins (i.e., AKT, MTOR, and FOXO3A) and primordial follicle atresia and apoptosis [29].

Another indirect effect of chemotherapy on the ovaries is exerted through stromal damage which occurs following stromal vessels damage and obstruction with subsequent stromal ischemia and fibrosis with cortical neovascularization and collagen deposition. Cortical neovascularization and collagen deposition lead to primordial follicle atresia [30].

The direct effect of chemotherapy on ovaries is exerted through direct ototoxicity and apoptosis. Chemotherapy breaks the double stranded DNA which leads to direct primordial follicle injury [31].

Cyclophosphamide as an example activates PTEN/PI3K/AKT pathways, which causes waves of premature ovarian follicle development with subsequent apoptosis rather than mature

ovarian follicle development. Regardless of the total dose of chemotherapy, continuous daily dose is more risky than single dose [32].

2. Radiotherapy: Radiation could accelerate the ovarian aging process leading to amenorrhea, and infertility. Granulosa cells which are the functional unit of the growing ovarian follicle are the main target during radiation. Radiation can cause significant depletion in the ovarian follicles pool within hours. Radiation damages the ovarian stroma blood supply and causes stromal damage, atrophy and fibrosis [33].

3. Gynecological surgeries: About 17-40% of endometriosis and 35% of benign ovarian cyst are presented with a clinical picture which necessitates surgical interventions . Laparoscopic approach is commonly used to treat pelvic pathologies such as ovarian cyst, fibroid and endometrioma [34].

Surgical excision of an ovarian cyst and/or endometrioma has an impact on the ovarian reserve. A significant decline in AMH was observed three days after an ovarian surgery [35]. A systematic review [36] found bipolar electrocoagulation can significantly harm ovarian reserve, particularly for women seeking fertility.

Owczarek et al. [37] found all hemostatic methods used during surgical removal of endometriotic cysts, resulted in diminished ovarian reserve.

A systematic review [38] suggested that cystectomy (especially bilateral) may not provide any reproductive benefits and could lead to diminished ovarian reserve.

The exact cause of diminished ovarian reserve after endometrioma excision remains unclear. It is possible that both surgical trauma and residual implants within the ovary contribute to this outcome. Considering these findings, conservative treatment may be a suitable first-line approach for women seeking reproductive options [39].

Autoimmune Disorders: Ovaries are usually affected during immune attacks with subsequent decreased ovarian reserve, decreased AMH and POF.

1. Systemic lupus erythematosus (SLE): Is an autoimmune disease characterized by generation of autoantibodies that may adversely affect ovarian reserve and fertility. SLE women of reproductive age have lower ovarian reserve compared to normal controls [40].

2. Diabetes Mellitus (DM) type-1: Ovarian follicles may be damaged directly by hyperglycemia and/or indirectly by the oxidative of DM [41].

3. Thyroid disorders: Is the most common reproductive age endocrine disorder with (i.e., 5-10% prevalence) [42].

The untreated hypothyroidism could lead to menstrual irregularities and ovarian follicles damage [43]. This could be explained by the presence of thyroid receptors in the ovary and ovarian damage in Hashimoto's thyroiditis. Additionally, women with thyroid disorders are at risks of anovulation, amenorrhea, menstrual irregularities and infertility [44].

Radio-frequency waves: Currently there is a dramatic increase in exposure to radio-frequency waves generated from cellular phones, wireless network technology (i.e., wireless fidelity or Wi-Fi), satellite and microwaves. Six billion people are currently using mobile phones, with a proportional increase in malignancy rates following increased harmful oxidative stress rates [45]. The difference between microwave and Wi-Fi signals lies in their radiation patterns and directionality: a typical microwave uses omnidirectional radiation, sending its signal outward in every possible direction, and uses more power than a Wi-Fi router (1000 watts). The standard Wi-Fi router sends its signal using only one direction (radiating from your device's nearest point of contact). A Wi-Fi router typically generates≤100 milliwatts (15-20 milliwatts for a standard Wi-Fi router). Wi-Fi waves could have direct adverse effect on endometrial tissue, and ovarian granulosa cells (quality and quantity) with subsequent abnormal folliculogenesis, and spontaneous miscarriages [46].

Obesity: Around 30% of reproductive age women had BMI between 25-30 kg/m^2 and 20% of them are obese. Obese women are at risk of reproductive problem, and lower fertility rate even those with regular menses [47].

Although, Fedorcsák et al. [48] found obese women during IVF programs are at risks of higher ovulation induction doses, fewer retrieved oocytes and higher failure rate.

Martinuzzi et al. [49] found obese did not affect the assisted reproductive techniques (ART) outcome. AMH is low in obese women compared to non-obese controls. Obesity related endocrine disorders may have a negative impact on the ovulation and ovarian reserve [50]. Moreover, obesity is associated with upregulation of the enzymes involved in androgen metabolism

with subsequent hyperandrogenism which inhibits the HPO through negative feedback mechanism [50].

Eating Habits: Junk food has high amounts of fully saturated fat and sugar with deficient minerals and vitamins [51,52]. High caloric intake in junk food is connected to obesity and subsequent PCOS (38-88% of PCOS women are overweight or obese) [53]. PCOS obese women are at risk of hyperandrogenism, irregular menses and infertility [54].

Endocrine-disrupting chemicals (EDCs): Are exogenous agents with the ability to disrupt and/or interfere with the endogenous hormones. EDCs can disrupt the ovarian hormonal function with subsequent anovulation, infertility, and POF through altering the ovarian hormones availability or altering the ovarian hormones bindings/activity at their receptors. EDCs include pesticides (i.e., dichlorodiphenyltrichloroethane), plasticizers (i.e., bisphenol A and phthalates), dioxins, polychlorinated biphenyls, aromatic hydrocarbons (i.e., benzopyrene) [55].

High intensity focused ultrasound (HIFU): Uterine fibroids (commonest benign tumor of uterus) are often presented with abnormal uterine bleeding, dysmenorrhea and/or pelvic pressure symptoms [56].

HIFU is a non-invasive method that uses external ultrasound energy that generates thermal ablation and thermos-coagulation to treat uterine fibroids (HIFU can be done through ultrasound or MRI guidance) [56].

Otonkoski et al. [57] and Qu et al. [58] found HIFU used for treating uterine fibroids and/or adenomyosis has no effect on ovarian reserve.

Conclusion: This review identified new causes/risks of POR (i.e., Wi-Fi waves and EDCs). The effect of these new causes/risks on the ovarian reserve needs future studies and needs an effective preventive strategy.
