# Supplementary material for: The correlation between serum vitamin D with Apo B and framingham risk score among a group of Iraqi subjects: a Cross-sectional and prospective pilot study
Source: BMC Cardiovasc Disord. 2025 Jul 3;25:445. doi: 10.1186/s12872-025-04855-w (PMC12224530; doi:10.1186/s12872-025-04855-w)
Supplement: Supplementary file 3 — Supplementary Material 3 [file 12872_2025_4855_MOESM3_ESM.pdf]

Jawatankuasa Etika
Penyelidikan Manusia USM (JEPeM)

Human Research Ethics Committee USM (HREC)

Universiti Sains Malaysia
Kampus Kesihatan
16150 Kubang Kerian, Kelantan, Malaysia.
Tel. : +609 - 767 3000/2354/2362
Fax. : + 609 - 767 2351
Email : jepem@usm.my
Laman Web : www.jepem.kk.usm.my
www.usm.my

31st October 2022

Mdm. Israa Nathir Almitwali
School of Pharmaceutical Sciences
Universiti Sains Malaysia
11800 Gelugor, Pulau Pinang

JEPeM Code : USM/JEPeM/22060348
Protocol Title: Correlation of Apolipoprotein B and Vitamin D Deficiency and Their Impact on Cardiovascular Risk Score

Dear Mdm.,

We wish to inform you that your study protocol has been reviewed and is hereby granted approval for implementation by the Jawatankuasa Etika Penyelidikan Manusia Universiti Sains Malaysia (JEPeM-USM). Your study has been assigned study protocol code **USM/JEPeM/22060348**, which should be used for all communications to JEPeM-USM in relation to this study. This ethical approval is valid from **31st October 2022** until **30th October 2023.**

Study Site: Private outpatient Clinical Laboratories of Dr. Abas Abd Almuaed, Al-Harthis street and Clinical Laboratories of Al-Nukhba, Baghdad (Iraq)

The following researchers are also involved in this study:
1. Dr. Fatimatuzzahra' Abd. Aziz
2. Dr. Raid Dhia Hashim

The following document has been approved for use in the study:
1. Research Proposal

In addition to the abovementioned document, the following technical documents were included in the review on which this approval is based:
1. Participant Information Sheet and Consent Form (English version)
2. Participant Information Sheet and Consent Form (Arabic version)
3. Data Collection Table

The list of JEPeM-USM members present during the full board meeting reviewing your protocol is attached.

While the study is in progress, we request that you submit to us the following documents:

1. Application for renewal of ethical approval 60 days before the expiration date of this approval through submission of **JEPeM-USM FORM 3(B) 2019: Continuing Review Application Form.**
2. Any changes in the protocol, especially those that may adversely affect the safety of the participants during the conduct of the trial including changes in personnel, must be submitted or reported using **JEPeM-USM FORM 3(A) 2019: Study Protocol Amendment Submission Form.**
3. Revisions in the informed consent form using the **JEPeM-USM FORM 3(A) 2019: Study Protocol Amendment Submission Form.**

4. Reports of adverse events including from other study sites (national, international) using the **JEPeM-USM FORM 3(G) 2019: Adverse Events Report.**
5. Notice of early termination of the study and reasons for such using **JEPeM-USM FORM 3(E) 2019.**
6. Any event which may have ethical significance.
7. Any information which is needed by the JEPeM-USM to do ongoing review.
8. Notice of time of completion of the study using **JEPeM-USM FORM 3(C) 2019: Final Report Form.**

Please note that forms may be downloaded from the JEPeM-USM website: www.jepem.kk.usm.my

JEPeM-USM is in compliance with the Declaration of Helsinki, International Conference on Harmonization (ICH) Guidelines, Good Clinical Practice (GCP) Standards, Council for International Organizations of Medical Sciences (CIOMS) Guidelines, World Health Organization (WHO) Standards and Operational Guidance for Ethics Review of Health-Related Research and Surveying and Evaluating Ethical Review Practices, EC/IRB Standard Operating Procedures (SOPs), and Local Regulations and Standards in Ethical Review.

Thank you.

"WAWASAN KEMAKMURAN BERSAMA 2030"

"BERKHIDMAT UNTUK NEGARA"

Sincerely,

Narazah

PROF. DR. NARAZAH MOHD YUSOFF
Advisor
Jawatankuasa Etika Penyelidikan (Manusia) JEPeM
Universiti Sains Malaysia

Jawatankuasa Etika
Penyelidikan Manusia USM (JEPeM)

Human Research Ethics Committee USM (HREC)

Universiti Sains Malaysia
Kampus Kesihatan
16150 Kubang Kerian, Kelantan, Malaysia.
Tel. : +609 - 767 3000/2354/2362
Fax. : + 609 - 767 2351
Email : jepem@usm.my
Laman Web : www.jepem.kk.usm.my
www.usm.my

Date of meeting : 19th October 2022
Venue : Through WEBEX Application
Time : 9.00 a.m – 1.00 p.m
Meeting No : 564

Members of the Jawatankuasa Etika Penyelidikan (Manusia), JEPeM Universiti Sains Malaysia who reviewed the protocol/documents are as follows:

Member (Title and Name)		Occupation (Designation)	Male/ Female (M/F)	Tick (✓) if present when above items, were reviewed
**Advisor:** Prof. Dr. Narazah Mohd Yusoff		Advisor of Jawatankuasa Etika Penyelidikan (Manusia), JEPeM USM	F	✓ **(Advisor)**
**Deputy Chairperson:** Assoc. Prof. Dr. Shahrom Mahmud		Deputy Chairperson of Jawatankuasa Etika Penyelidikan (Manusia), JEPeM USM	M	✓ **(Deputy Chairperson)**
**Member Secretary:** Dr. Nik Norma Nik Hasan		Member Secretary of Jawatankuasa Etika Penyelidikan (Manusia), JEPeM USM	F	✓ **(Member Secretary)**
**Secretariat:** Mdm. Nor Amira Khurshid Ahmed		Research Officer	F	✓
**Members :**				
1.	Mr. Abdul Hamid Ibrahim Naina	Community Representatives	M	✓
2.	Mr. Anuar Omar	Community Representatives	M	✓
3.	Assoc. Prof. Dr. Garry Kuan Pei Ern	Lecturer, School of Health Sciences	M	✓
4.	Dr. Goh Yen Nee	Lecturer, Graduate School of Business	F	✓
5.	Dr. Hadzliana Zainal	Lecturer, School of Pharmaceutical Sciences	F	✓
6.	Dr. Izham Mohamad Yusoff	Lecturer, School of Distance Education	M	✓
7.	Assoc. Prof. Dr. Loke Yiing Jia@ Loke Weng Kah	Lecturer, School of Social Sciences	F	✓
8.	Dr. Maizura Murad	Lecturer, School of Industrial Technology	F	✓

Jawatankuasa Etika Penyelidikan (Manusia), JEPeM-USM is in compliance with the Declaration of Helsinki, International Conference on Harmonization (ICH) Guidelines, Good Clinical Practice (GCP) Standards, Council for International Organizations of Medical Sciences (CIOMS) Guidelines, World Health Organization (WHO) Standards and Operational Guidance for Ethics Review of Health-Related Research and Surveying and Evaluating Ethical Review Practices, EC/IRB Standard Operating Procedures (SOPs), and Local Regulations and Standards in Ethical Review.

Narazah

PROF. DR. NARAZAH MOHD YUSOFF
Advisor
Jawatankuasa Etika Penyelidikan (Manusia), JEPeM
Universiti Sains Malaysia

JAWATANKUASA ETIKA PENYELIDIKAN MANUSIA
