# Supplementary material for: The correlation between serum vitamin D with Apo B and framingham risk score among a group of Iraqi subjects: a Cross-sectional and prospective pilot study
Source: BMC Cardiovasc Disord. 2025 Jul 3;25:445. doi: 10.1186/s12872-025-04855-w (PMC12224530; doi:10.1186/s12872-025-04855-w)
Supplement: Supplementary file 4 — Supplementary Material 4 [file 12872_2025_4855_MOESM4_ESM.pdf]

يو إس إم (جامعة العلوم الماليزية) - أبيكس

لجنة أخلاقيات البحوث (الانسانية) "JEPeM USM"
جامعة العلوم الماليزية

نموذج صحيفة معلومات المشارك واستمارة الموافقة

(مشروع بحث)

1. الملحق "ب"
عنوان البحث: العلاقة بين نقص صميم البروتين الشحمي "ب" وفيتامين "د"، وتأثيرهما على درجة مخاطر الإصابة بأمراض القلب والأوعية الدموية.

2. الملحق "ق" (معلومات المشترك واستمارة الموافقة)

3. الملحق "ع" (استمارة الموافقة على نشر المواد الخاصة بالمشترك)

المترجم القانوني
جهاد الطائي
عضو جمعية المترجمين العراقيين
رقم الهوية 1854

مثال　　　　**الملحق "ب"**

معلومات البحث

عنوان البحث: العلاقة بين نقص صميم البروتين الشحمي "ب" وفيتامين "د"، وتأثيرهما على درجة مخاطر الإصابة بأمراض القلب والأوعية الدموية.
الاسم الرئيسي: اسراء ناثر (P-FD0037/21(R))
الباحث المساعد: د. فاطمة الزهراء بنت عبد العزيز، د. رائد ضياء هاشم
مقدمة
إنّ المشتركين في المرحلة 1 من الدراسة الذين يعانون من نقصٍ حادٍ في فيتامين "د" مدعوون للمشاركة الطوعية في دراسة تداخلية (تجريبية) (المرحلة 2)، حيث سيُطلب من المشترك تناول دورة علاجيةٍ من فيتامين "د" من أجل تصحيح حالة فيتامين "د" لديهم (مجموعة العلاج) وهذا سيتطلب (8) ثمانية أسابيع من تناول تركيز (50,000) وحدة دولية من فيتامين "د" تليها جرعة يومية تركيز (1,000) وحدة دولية لمدّة أربعة أشهر؛ في حين يُعتبر الأشخاص ذوي المستوى العادي من فيتامين "د" كمجموعة الضبط (لتقديم عيّنة دم ثانية من أجل فحص مستوى فيتامين "د" وصميم البروتين الشحمي "ب" بنهاية الستة أشهر). في الشهر السادس، سيتم جمع عينات دم (5) مل من الوريد الأمامي لتقييم تأثير تصحيح مستوى فيتامين "د" على مستويات صميم البروتين الشحمي "ب".

يتمحور هذا البحث حول تحديد تأثير تصحيح مستويات فيتامين "د" على مستويات صميم البروتين الشحمي "ب".

من المهم أن تقرأ وتفهم معلومات البحث هذه قبل الموافقة على المشاركة في هذه الدراسة. إذا وافقتَ على المشاركة، فسيتم تسليمك نسخةً من هذه الاستمارة لتحفظها في سجلاتك.

من المتوقع أن تستغرق مشاركتك في هذه الدراسة مدة عشر دقائق لجمع عيّنات دم. وعليك تناول فيتامين "د" على النحو الموصوف من قبل الطبيب لمدّة (6) ستة أشهر لتصحيح مستوى فيتامين "د" لديك (فقط لمجموعة العلاج). من المقدّر أن تشتمل هذه الدراسة على ما يصل إلى (59) مشتركاً مصاباً بنقصٍ حادٍ في فيتامين "د" بصفتهم مجموعة العلاج و(40) مشتركاً ذوي مستوى عادي من فيتامين "د" بصفتهم مجموعة الضبط.

هدف الدراسة
تهدف هذه الدراسة إلى اكتشاف تأثير تصحيح مستوى فيتامين "د" على مستويات صميم البروتين الشحمي "ب".

معايير المشاركين
سيتولى أعضاء فريق البحث مناقشة أهليتك للمشاركة في هذه الدراسة. من الهام جداً أن تكون صادقاً بشكلٍ كاملٍ مع الكادر بما في ذلك تاريخك الصحي والدوائي.

تشتمل هذه الدراسة (معايير الشمول) على الأفراد البالغين الذين أعمارهم (≤20) عاماً ويراجعون المختبرات السريرية الثلاث للمرضى الخارجيين لمدّة (6) أشهر – من تشرين الأول 2022 لغاية 2023. سيُطلب من المرضى المصابين بنقصٍ حادٍ في فيتامين "د" (< 10 نانوغرام/مل) للمتابعة للمرحلة 2 في مجموعة العلاج، في حين أنّ أصحاب المستويات العادية من فيتامين "د" (≤30 نانو غرام/مل) يُعتبرون مجموعة الضبط.

لن تتضمن هذه الدراسة (معايير الاستبعاد) الأفراد الذين رفضوا مشاركة بياناتهم والأفراد المصابين بأمراض تصلب الشرايين أو أيّ أمراضٍ مزمنةٍ أو أمراضٍ حادةٍ حديثة (قبل 2-3 أشهر) أو الأمراض الأقل حدّة (2-3 أسابيع) أو الذين يتلقون العلاج باستثناء ارتفاع ضغط الدم ومستويات فيتامين "د" (10-30 نانو غرام/مل والنساء الحوامل والمرضعات وأولئك الذين هم دون عمر (20) سنةً.

المترجم القانوني
جهاد الطائي
عضو جمعية المترجمين العراقيين
رقم الهوية 1854

بغداد – المنصور – قرب تقاطع 14 رمضان – مقابل مطعم بيستون صمد
Baghdad, Almansour
موبايل: Mobile: 07808585128 / 07710522456
Email: Jihadquaik@gmail.com

I, the sworn translator Jihad Al-Taee, certify hereby the validity and accuracy of translation without assuming any responsibility of its contents

معايير الانسحاب (المرحلة 1): بالنسبة للمشاركين الراغبين في إيقاف مشاركتهم بأيّ مرحلةٍ من مراحل الدراسة، سيتم السماح لهم بالانسحاب وسيتم استبعادهم من الدراسة والتحليل.

إجراءات الدراسة
سيتم اختيار مئة وعشرين مشاركاً بشكلٍ عشوائيٍ من بين قائمة الحضور للمختبر السرير من بين أولئك الذين أعطوا موافقتهم المستنيرة فقط للمشاركة في الدراسة. سيجري الاتصال بالمشتركين هاتفياً للاشتراك بالدراسة وتقديم موافقتهم المستنير ولأخذ عيّنات الدم لفحص المختبر. تُؤخذ مستويات السكر بالدم واختبارات وظائف الكبد والكلى من السجلات الطبية المختبرية واستخدامها للكشف عن الأمراض دون أعراضٍ. في حين سيتم الحصول على البيانات الديموغرافية وإجمالي نسبة الكولسترول والكوليسترول الدهني عالي الكثافة من السجلات الطبيّة. وسيجري أخذ عيّنات دم (5) مل من قِبل فنيي عالي المهارة لفحص المختبر من أجل الفحص المختبري لصميم البروتين الشحمي "ب" وفيتامين "د". بعد ذلك الانتقاء العشوائي لواحدٍ وخمسين مشاركاٍ (من ملف الاكسل الخاص بنتائج المرحلة 1) المصابين بنقصٍ حادٍ في فيتامين "د"، إذ سيُطلب منهم المشاركة في المرحلة 2 التداخلية (بصفتهم مجموعة العلاج) الخاصة بدراسة تصحيح فيتامين "د"، إضافةً إلى (40) مشتركاً ذوو مستويات عادية من فيتامين "د" (بصفتهم مجموعة الضبط). لهذا الجزء من الدراسة، ستكون أرقام الهاتف والبريد الالكتروني مطلوبةً لغرض المتابعة. سيكون الطبيب متاحاً لوصف جرعة فيتامين "د" (50,000 وحدة دولية/أسبوع) لمدّة ثماني أسابيع تليها جرعة (1,000) وحدة دولية يومياً لغاية الشهر 6 للمشاركين بالمصابين بنقصٍ حادٍ ولإدارة أي حالةٍ سريرية قد تتطور أثناء الدراسة.
هذه الجرعة من فيتامين "د" موصى بها من أطباء الأسرة الأمريكيين في عام 2009 لتصحيح نقص فيتامين "د". وسيكون كلٌ من مشاركي مجموعة العلاج والضبط مدعوون لتقديم عينات الدم الثانية (5 مل) من الوريد بواسطة فني مختبر عالي المهارة) بنهاية الشهر 6. سيستغرق وقت المشاركة 10 عشر دقائق فقط لأخذ عينات الدم وفحص مصل فيتامين "د" وصميم البروتين الشحمي "ب".
ستُعالج عينات الدم الزائدة كيميائياً أو بواسطة جهاز الاوتوكلاف، وتُجمع في حاوياتٍ خاصةٍ بشكلٍ يوميٍّ، ومن ثم سيقوم ممثلٌ للوزارة بجمع العيّنات الملوّنة للتخلص منها وفقاً لضوابط المختبرات العراقية. تُحمّل البيانات وتُخزن على قرص خارجي لتجنب ضياعها وضمان الحفاظ على السريّة. الباحثون فقط هم من يملكون صلاحية الوصول إلى تلك البيانات. لن يتم استخدام أي معلوماتٍ قابلةٍ للتحديد أثناء تحليل البيانات. يتم تحليل البيانات على حاسبٍ محمي بكلمة مرور. بعد استكمال الدراسة، سيتم حذف الملفات المؤقتة من الحاسوب وحفظ البيانات المشفّرة لمدة (3) ثلاث سنوات كحدٍ أدنى على قرصٍ خارجيٍّ محميٍّ بكلمة مرور، ومن ثم سيتم التخلّص منها. بعد الحذف الكلي للبيانات الالكترونية، سيتم اتلاف النسخة الصلبة الخارجية من البيانات، ولن يكون هناك أيّ نسخة صلبة عن البيانات.

المخاطر
لا يوجد أي مخاطرٍ محتملةٍ مثل التعرض للضغط النفسي للمشارك أو لشريكه.

المترجم القانوني
جهاد الطائي
عضو جمعية المترجمين العراقيين
رقم الهوية 1854

Baghdad, Almansour
Email: Jihadquaik@gmail.com
بغداد – المنصور – قرب تقاطع 14 رمضان – مقابل مطعم بيستون صمد
Mobile: 07808585128 / 07710522456 :موبايل

I, the sworn translator Jihad Al-Taee, certify hereby the validity and accuracy of translation without assuming any responsibility of its contents

الإبلاغ عن المشاكل الصحية
يرجى الاتصال بأيّ وقتٍ بالباحث المذكور تالياً إذا واجهت أي مشكلة صحية ترتبط بشكلٍ مباشرٍ أو غير مباشرٍ بهذه الدراسة.
د. اسراء ناثر [رقم تسجيل "MMC": P-FD0037/21(R) رقم الموبايل: +9647811918598]
د. رائد ضياء +9647827524118

المشاركة في الدراسة
إنّ اشتراكك في هذه الدراسة هو اشتراكٌ طوعيّ بالكامل. يمكنك رفض المشاركة في الدراسة أو التوقف عن المشاركة في أيّ وقتٍ دون التعرض لأيّ غرامةٍ أو خسارة أيّ امتيازاتٍ تستحقها خلاف ذلك. كذلك قد يتم إيقاف مشاركتك بواسطة فريق البحث دون استشارتك في حال خرقك لمعايير الأهلية الخاصة بالدراسة. سيقوم عضوٌ من فريق البحث بمناقشة ذلك الأمر حال حصوله. لديك الحق في رفض الاستخدام المستقبلي وتخزين العينات/البيانات التي تم جمعها.

الفوائد المحتملة [فوائد للأشخاص وللمجتمع وللجامعة]
لن يحصل المشاركون على مكاسب شخصية أو مزايا اقتصادية. ستحصل على دورة علاج مجاني لنقص فيتامين "د" (فقط لمن يشارك في المرحلة 2 من الدراسة).

لن تقدّم هذه الدراسة فائدة مباشرة للمشاركين، إلا أنّها ستقدّم منفعة للمجتمع حيث أنها ستضيف المزيد من الأدلة لتحسين وتحديث ضوابط تقييم مخاطر الإصابة بأمراض القلب والشرايين.
لن تحصل على أي مبالغٍ متوقعةٍ أو تعويضاتٍ من هذه الدراسة.
ولن يتم تقديم تعويضاتٍ لك أو لعائلتك في حالة الإعاقة أو الموت الناتج عن إصاباتٍ متعلقةٍ بالدراسة حيث تتبع الدراسة الإرشادات ولا يوجد أي مخاطرٍ متوقعةٍ للمشتركين.

الأسئلة
إن كان لديك أيّ استفساراتٍ حول هذه الدراسة أو حول حقوقك، يرجى الاتصال بـ:
د. اسراء ناثر [رقم تسجيل "MMC": P-FD0037/21(R)]
قسم الصيدلة السريرية
مدرسة العلوم الصيدلانية
الحرم الجامعي الرئيسي لجامعة العلوم الماليزية
+9647811918598

إذا كان لديك أسئلة حول الموافقة الأخلاقية أو حول أيّ مواضيعٍ/مشاكلٍ متعلقةٍ بهذه الدراسة، يرجى الاتصال بـ:

السيد محمد بازلان حافظ مكريم
أمين لجنة أخلاقيات البحوث الانسانية – جامعة العلوم الماليزية
قسم البحث والابتكار
الحرم الصحي لجامعة العلوم الماليزية
رقم الهاتف: 2362 767-09/ 2354 767-09
البريد الالكتروني: bazlan@usm.my
أو
الأنسة نور أميرة خورشيد احمد
أمانة لجنة أخلاقيات البحوث الانسانية – جامعة العلوم الماليزية
مكتب الإدارة والإبداع البحثي
الحرم الرئيسي لجامعة العلوم الماليزية، بينانغ
رقم هاتف: 04-6536537
البريد الالكتروني: noramira@usm.my

المترجم القانوني
جهاد الطائي
عضو جمعية المترجمين العراقيين
رقم الهوية 1854

السريّة
سيقوم الباحثين بالحفاظ على معلوماتك سريّةً من قِبَل ولن يتم اتاحتها علناً إلا إن كان الإفصاح عنها مطلوباً بمقتضى القانون.
إنّ المعلومات المستحصلة من هذه الدراسة والتي لا تُحدّد شخصيتك بشكل فردي ستُنشر لأغراض المعرفة.

سيتم مراجعة سجلاتك من قِبَل الباحثين ومجلس المراجعة الاخلاقية الخاص بهذه الدراسة والسلطات التنظيمية من أجل التحقق من إجراءات أو بيانات الدراسة. قد يتم حفظ ومعالجة معلوماتك على الحاسوب. يمتلك فقط أعضاء فريق البحث الحق في الوصول إلى معلوماتك.

عبر توقيع استمارة الموافقة هذه فإنّك تمنح الترخيص لمراجعة السجل وحفظ المعلومات ومعالجة البيانات المذكورة أعلاه.

التواقيع
لكي تدخل في الدراسة يجب أن تقوم أنت أو ممثلك القانوني بوضع التوقيع والتاريخ على صفحة التوقيع [الملحق "ق" أو الملحق "ع"]

المترجم القانوني
جهاد الطائي
عضو جمعية المترجمين العراقيين
رقم الهوية 1854

Baghdad, Almansour
Email: Jihadquaik@gmail.com
بغداد – المنصور – قرب تقاطع 14 رمضان – مقابل مطعم بيستون صمد
موبايل: 07808585128 / 07710522456 :Mobile

I, the sworn translator Jihad Al-Taee, certify hereby the validity and accuracy of translation without assuming any responsibility of its contents

الملحق "ق"

معلومات وموافقة المتقدّم
(صفحة التوقيع)

عنوان البحث: العلاقة بين نقص صميم البروتين الشحمي "ب" وفيتامين "د"، وتأثيرهما على درجة مخاطر الإصابة بأمراض القلب والأوعية الدموية.

اسماء القائمين بالبحث: اسراء ناثر احمد، د. فاطمة الزهراء بنت عبد العزيز، د. رائد ضياء هاشم

للاشتراك في هذه الدراسة، عليك أن تقوم أنت أو ممثلك القانوني بتوقيع هذه الصفحة. إنني أؤكّد عبر توقيع هذه الصفحة التالي:

- أنني قرأت كل المعلومات في استمارة معلومات المشترك و استمارة الموافقة هذه بما في ذلك المعلومات المتعلقة بمخاطر هذه الدراسة وكان لدي الوقت الكافي للتفكير بشأنها.
- تمت الإجابة على كافة أسئلتي بشكلٍ مقنع.
- أوافق طوعاً على أن أكون جزءاً من هذا البحث وعلى اتباع إجراءات الدراسة وتقديم المعلومات اللازمة إلى الطبيب أو الممرضة أو بقية أعضاء الكادر على النحو المطلوب.
- امتلك الحرية في اختيار التوقف عن المشاركة في هذه الدراسة في أيّ وقتٍ.
- لقد استلمت نسخةً من استمارة معلومات وموافقة المشترك هذه لأحتفظ بها لنفسي.

اسم المشترك

رقم بطاقة تعريف المشترك

توقيع المشترك أو الممثل القانوني — التاريخ (يوم/شهر/سنة)

اسم الشخص
الذي أجرى مناقشة الموافقة

توقيع الشخص
الذي أجرى مناقشة الموافقة — التاريخ (يوم/شهر/سنة)

اسم وتوقيع الشاهد — التاريخ (يوم/شهر/سنة)

ملاحظة: 1) جميع المشتركين في هذه الدراسة لن يتم تغطيتهم بالتأمين.

المترجم القانوني
جهاد الطائي
عضو جمعية المترجمين العراقيين
رقم الهوية 1854

Baghdad, Almansour
بغداد – المنصور – قرب تقاطع 14 رمضان – مقابل مطعم بيستون صعد
Email: Jihadquaik@gmail.com
Mobile: 07808585128 / 07710522456 :موبايل

I, the sworn translator Jihad Al-Taee, certify hereby the validity and accuracy of translation without assuming any responsibility of its contents

الملحق "ع"

استمارة الموافقة على نشر المواد الخاصة بالمشترك
صفحة التوقيع

عنوان البحث: العلاقة بين نقص صميم البروتين الشحمي "ب" وفيتامين "د"، وتأثيرهما على درجة مخاطر الإصابة بأمراض القلب والأوعية الدموية.

اسماء القائمين بالبحث: اسراء ناثر احمد، د. فاطمة الزهراء بنت عبد العزيز، د. رائد ضياء هاشم

لتكون جزءاً من هذه الدراسة، عليك أن تقوم أنت أو ممثلك القانوني بالتوقيع على هذه الورقة.
إنّني أؤكد من خلال توقيعي على هذه الصفحة ما يلي:

- أفهم بأن اسمي لن يظهر على المواد المنشورة وبأنّ هناك جهوداً مبذولةً للتأكّد من حفظ سريّة اسمي بالرغم من أن السريّة غير مضمونة بالكامل في ظل الظروف غير المتوقعة.
- لقد قرأت المواد أو الوصف العام حول مضمون المواد وراجعت كل الصور التي أتواجد فيها والتي قد يتم نشرها.
- لقد أُتيحت لي الفرصة لقراءة النص ورؤية كافة المواد التي تشتمل على ذكري، إلا أنني تنازلت عن حقي في القيام بذلك.
- سيتم مشاركة كل المواد المطبوعة بين ممارسي الطب والعلماء والصحفيين في أنحاء العالم.
- كذلك ستُستخدم تلك المواد في المطبوعات المحلية والكتب وسيتمكن العديد من الأطباء المحليين والعالميين من الوصول إليها.
- أوافق بموجبه وأسمح باستخدام المواد في منشوراتٍ أخرى مطلوبة بواسطة ناشرين آخرين بموجب الشروط التالية:
  - عدم استخدام المواد لأغراض الدعاية أو كمواد تغليفٍ.
  - لن تُستخدم المواد خارج السياق. بعبارةٍ أخرى: صور العينات لن تُستخدم في مقالةٍ ذات موضوعٍ غير مرتبطٍ بالصورة.

اسم المشترك ____________________

رقم هوية المشترك ____________________ توقيع المشترك ____________________ التاريخ (يوم/شهر/سنة) ____________________

اسم وتوقيع الشخص الذي أجرى مناقشة الموافقة ____________________ التاريخ (يوم/ شهر/ سنة) ____________________

ملاحظة: 1) جميع المشتركين في هذه الدراسة لن يتم تغطيتهم بالتأمين.

المترجم القانوني
جهاد الطائي
عضو جمعية المترجمين العراقيين
رقم الهوية 1854

بغداد – المنصور – قرب تقاطع 14 رمضان – مقابل مطعم بيستون صمد
موبايل: Mobile: 07808585128 / 07710522456
Baghdad, Almansour
Email: Jihadquaik@gmail.com

I, the sworn translator Jihad Al-Taee, certify hereby the validity and accuracy of translation without assuming any responsibility of its contents

JAWATANKUASA ETIKA PENYELIDIKAN (MANUSIA) – JEPeM USM
UNIVERSITI SAINS MALAYSIA

TEMPLATE OF PARTICIPANT INFORMATION SHEET AND CONSENT FORM

(RESEARCH PROJECT)

1. ATTACHMENT B

RESEARCH TITLE: Correlation of Apolipoprotein B and Vitamin D Deficiency and Their Impact on Cardiovascular Risk Score

2. ATTACHMENT S (Participant Information and Consent Form)

3. ATTACHMENT P (Participant's Material Publication Consent Form)

RESEARCH INFORMATION

Research Title: Correlation of Apolipoprotein B and Vitamin D Deficiency and Their Impact on Cardiovascular Risk Score
Name of main: Israa Nather (P-FD0037/21(R))
Co-Researcher: Dr. Fatimatuzzahra' Binti Abd. Aziz, Dr. Raid Dhia Hashim

INTRODUCTION

Participants of phase 1 of the study with severe vitamin D deficiency are invited to take part voluntarily in an interventional (experimental) study (phase 2) as he/she will be asked to take a course of vitamin D in order to correct their vitamin D status (treatment group), as this will need 8 weeks of 50000 IU then 1000 IU daily for four months and those with normal vitamin D will be considered as a control group (to give a second blood sample to examine their vitamin D and apolipoprotein B levels at the end of 6 months). At month 6 blood samples of 5 ml from the antecubital vein will be collected to assess the effect of vitamin D correction on Apolipoprotein B levels

This research is about the determination of the effect of correcting vitamin D levels on apolipoprotein B levels.

It is important that you read and understand this research information before agreeing to participate in this study. You will receive a copy of this form to keep for your records if you agree to participate.

Your participation in this study is expected to take 10 minutes for blood sample collection and you should take vitamin D as it will be prescribed by the physician for 6 months to correct your vitamin D level (for the treatment group only). This study is estimated to include up to 59 participants with severe vitamin D deficiency as a treatment group and 40 participants with normal vitamin D as a control group.

PURPOSE OF THE STUDY

The purpose of this study is to explore the effect of vitamin D correction on Apolipoprotein B levels.

PARTICIPANTS CRITERIA

The research team members will discuss your eligibility to participate in this study. It is important that you are completely truthful with the staff including your health and medication history.
This study will include (Inclusion Criteria) individuals who are adults aged ≥20 years old attending the three private outpatient clinical laboratories for a period of 6 months, from October 2022 to March 2023. Patients with severe vitamin D deficiency (< 10ng/ml) will be asked to continue for phase 2 in the treatment group of the study and those with normal vitamin D (≥30 ng/ml) will be considered in the control group.

This study will not include (Exclusion Criteria) individuals who refused to share their data, patients with CVDs, any chronic illness, recent acute severe illness (2-3 months before), recent acute minor illness (2-3 weeks), and taking treatment except for hypertension, vitamin D levels (10-30ng/ml), pregnant and breastfeeding women, also those under 20 years old.

Withdrawal Criteria (phase 2): participants who want to stop their participation at any stage of the study, they will be allowed to withdraw and they will be excluded from the study and the analysis.

STUDY PROCEDURES

A random selection of 120 participants from the list of attendance of the clinical laboratory for those who gave their informed consent only will be invited to join the study. Participants will be contacted by telephone to join the study, to give their informed consent, and for blood sampling for laboratory examination. Fasting blood glucose, liver, and kidney function tests will be taken from the laboratory medical records and used as a screening for any asymptomatic diseases. Demographic data, total cholesterol, and high-density lipoprotein cholesterol will be obtained from the medical records. Blood samples of 5 ml from the antecubital vein will be taken by a highly skilled technician for laboratory examination for apolipoprotein B, and vitamin D. After that random selection of fifty-nine participants (from the excel sheet of the results of phase 1) with severe vitamin D deficiency will be asked to join the interventional phase 2 (as a treatment group) of the study for vitamin D correction and 40 participants with normal vitamin D levels as a control group. For this part of the study, telephone numbers and emails of participants will be requested for follow-up. A physician will be available to prescribe vitamin D 50000 IU/week for 8 weeks then 1000 IU daily until month 6 to participants with severe deficiency and to manage any clinical condition that may develop during the study. This dose of vitamin D is recommended by American family physicians in 2009 for vitamin D deficiency correction. Both treatment and the control group participants will be invited to give 2nd blood sample **(5 ml from the antecubital vein by a highly skilled laboratory technician)** at the end of month 6. Participation time will be only 10 minutes for blood sampling to examine serum vitamin D and apolipoprotein B. **The excess blood specimen will be treated chemically or by an autoclave and collected in special containers daily then a representative of the ministry of health will collect the tainted samples to dispose them of according to the Iraqi laboratory guidelines. The data will be downloaded and stored on an external drive to avoid their loss and to ensure confidentiality. Only the researcher will have access to the data. No identifiable information will be used during data analysis. The data will be analyzed on a password-protected computer. After the accomplishment of the study, all temporary files will be deleted from the computer and the encrypted data will be maintained for a minimum period of 3 years in the password-protected external drive and will thereafter be disposed of. After completely deleting the electronic data, the external hard will be physically damaged. There will be no hard copy of the data.**

RISKS

No potential possible risk e.g. psychological distress to the participants or to his/ her partner or family in the treatment and the control group.

REPORTING HEALTH EXPERIENCES.

Please contact, at any time, the following researcher if you experience any health problem either directly or indirectly related to this study.
Dr. Israa Nathir **[MMC Registration No.** P-FD0037/21(R) at +9647811918598
Dr. Raid Dhia +9647827524118

PARTICIPATION IN THE STUDY

Your taking part in this study is entirely voluntary. You may refuse to take part in the study or you may stop your participation in the study at any time, without any penalty or loss of benefits to which you are otherwise entitled. Your participation also may be stopped by the research team without your consent if in any form you have violated the study eligibility criteria. The research team member will discuss it with you if the matter arises. **<u>You have the right to refuse future use and storage of the collected specimen/data.</u>**

POSSIBLE BENEFITS [Benefit to Individual, Community, University]

Participants won't get any personal benefit or, economic advantage. They will get a free treatment course for vitamin D deficiency (only for those who will join phase 2 of the study)
This study will not directly benefit the participants but it will benefit the community as it will add more evidence to improve and update guidelines in assessing CVD risk
<u>You will not receive any anticipated payments or compensation from this study</u>.
No compensation for you or your family in case of disability or death resulting from study-related injuries as the study follows the guidelines and no expected hazard to participants.

QUESTIONS

If you have any questions about this study or your rights, please contact;

Dr. Israa Nathir [MMC Registration No. P-FD0037/21(R)
Department of Clinical Pharmacy
School of Pharmaceutical Sciences
USM Main Campus
+9647811918598

If you have any questions regarding the Ethical Approval or any issue/ problem related to this study, please contact;

Mr. Mohd Bazlan Hafidz Mukrim
Secretary of Human Research Ethics Committee USM
Division of Research & Innovation (R&I)
USM Health Campus
Tel. No. : 09-767 2354 / 09-767 2362
Email : bazlan@usm.my

OR

Miss Nor Amira Khurshid Ahmed
Secretariat of Human Research Ethics Committee USM
Research Creativity & Management Office (RCMO)
USM Main Campus, Penang
Tel. No. : 04-6536537
Email : noramira@usm.my

CONFIDENTIALITY

Your information will be kept confidential by the researchers and will not be made publicly available unless disclosure is required by law.

Data obtained from this study that does not identify you individually will be published for knowledge purposes.

Your original records may be reviewed by the researcher, the Ethical Review Board for this study, and regulatory authorities for the purpose of verifying the study procedures and/or data. Your information may be held and processed on a computer. Only research team members are authorized to access your information.

By signing this consent form, you authorize the record review, information storage and data process described above.

SIGNATURES

To be entered into the study, you or a legal representative must sign and date the signature page **[ATTACHMENT S or ATTACHMENT P]**

Subject Information and Consent Form
(Signature Page)

Research Title: Correlation of Apolipoprotein B and Vitamin D Deficiency and Their Impact on Cardiovascular Risk Score

Researcher's Name: Israa Nathir Ahmed, Dr. Fatimatuzzahra' Binti Abd. Aziz, Dr. Raid Dhia Hashim

To become a part of this study, you or your legal representative must sign this page. By signing this page, I am confirming the following:

- I have read all of the information in this Patient Information and Consent Form **including any information regarding the risk in this study** and I have had time to think about it.
- All of my questions have been answered to my satisfaction.
- I voluntarily agree to be part of this research study, to follow the study procedures, and to provide necessary information to the doctor, nurses, or other staff members, as requested.
- I may freely choose to stop being a part of this study at any time.
- I have received a copy of this Participant Information and Consent Form to keep for myself.

____
Participant Name

____
Participant I.C No

____ ______________________
Signature of Participant or Legal Representative **Date** (dd/MM/yy)

____
Name of Individual
Conducting Consent Discussion

____ ______________________
Signature of Individual **Date** (dd/MM/yy)
Conducting Consent Discussion

____ ______________________
Name & Signature of Witness **Date** (dd/MM/yy)

Note: i) All participants who are involved in this study will not be covered by insurance.

Participant's Material Publication Consent Form
Signature Page

Research Title: Correlation of Apolipoprotein B and Vitamin D Deficiency and Their Impact on Cardiovascular Risk Score

Researcher's Name: Israa Nather, Dr. Fatimatuzzahra' Binti Abd. Aziz, Dr. Raid Dhia Hashim

To become a part of this study, you or your legal representative must sign this page.
By signing this page, I am confirming the following:

- I understood that my name will not appear on the materials published and there have been efforts to make sure that the privacy of my name is kept confidential although the confidentiality is not completely guaranteed due to unexpected circumstances.
- I have read the materials or general description of what the material contains and reviewed all photographs and figures in which I am included that could be published.
- I have been offered the opportunity to read the manuscript and to see all materials in which I am included, but have waived my right to do so.
- All the published materials will be shared among medical practitioners, scientists, and journalists worldwide.
- The materials will also be used in local publications, and book publications and accessed by many local and international doctors worldwide.
- I hereby agree and allow the materials to be used in other publications required by other publishers with these conditions:
- The materials will not be used for advertising purposes nor as packaging materials.
- The materials will not be used out of context – i.e.: Sample pictures will not be used in an article that is unrelated subject to the picture.

____

Participant Name

__________________	____________________	____________________
**Participant I.C No.**	**Participant's Signature**	**Date** (dd/MM/yy)

____	____________________
**Name and Signature of Individual** Conducting Consent Discussion	**Date** (dd/MM/yy)

Note: i) All participants who are involved in this study will not be covered by insurance.
